# Progress in Active Infrared Imaging for Defect Detection in the Renewable and Electronic Industries

**DOI:** 10.3390/s23218780

**Published:** 2023-10-27

**Authors:** Xinfeng Zhao, Yangjing Zhao, Shunchang Hu, Hongyan Wang, Yuyan Zhang, Wuyi Ming

**Affiliations:** 1 College of Water Conservancy Engineering, Yellow River Conservancy Technical Institute, Kaifeng 475000, China; zhaoxinfeng@yrcti.edu.cn; 2 Henan Key Laboratory of Intelligent Manufacturing of Mechanical Equipment, Zhengzhou University of Light Industry, Zhengzhou 450002, China; zhaoyj0529@163.com (Y.Z.); hushunchang2022@gmail.com (S.H.); hongyanwang923@163.com (H.W.); 3 Guangdong Provincial Key Laboratory of Digital Manufacturing Equipment, Guangdong HUST Industrial Technology Research Institute, Dongguan 523808, China

**Keywords:** infrared thermographic, renewable industry, electronic industry, algorithms, artificial intelligence

## Abstract

In recent years, infrared thermographic (IRT) technology has experienced notable advancements and found widespread applications in various fields, such as renewable industry, electronic industry, construction, aviation, and healthcare. IRT technology is used for defect detection due to its non-contact, efficient, and high-resolution methods, which enhance product quality and reliability. This review offers an overview of active IRT principles. It comprehensively examines four categories based on the type of heat sources employed: pulsed thermography (PT), lock-in thermography (LT), ultrasonically stimulated vibration thermography (UVT), and eddy current thermography (ECT). Furthermore, the review explores the application of IRT imaging in the renewable energy sector, with a specific focus on the photovoltaic (PV) industry. The integration of IRT imaging and deep learning techniques presents an efficient and highly accurate solution for detecting defects in PV panels, playing a critical role in monitoring and maintaining PV energy systems. In addition, the application of infrared thermal imaging technology in electronic industry is reviewed. In the development and manufacturing of electronic products, IRT imaging is used to assess the performance and thermal characteristics of circuit boards. It aids in detecting potential material and manufacturing defects, ensuring product quality. Furthermore, the research discusses algorithmic detection for PV panels, the excitation sources used in electronic industry inspections, and infrared wavelengths. Finally, the review analyzes the advantages and challenges of IRT imaging concerning excitation sources, the PV industry, the electronics industry, and artificial intelligence (AI). It provides insights into critical issues requiring attention in future research endeavors.

## 1. Introduction

Any object in nature that is above absolute temperature (−273 °C) radiates heat (electromagnetic waves) outward [1]. Electromagnetic waves with a wavelength range of 760 nm to 1 mm are called infrared and cannot be seen by the naked eye. The higher the temperature of an object, the greater the energy radiated. Infrared thermographic (IRT) technology involves sensing infrared waves through special materials, converting them into electrical signals, and then converting the electrical signals into digital images. Using thermal imaging technology, the detection device (an infrared thermal imager) receives varying degrees of infrared radiation from the surface of a sample, generating a temperature field map. This temperature field map characterizes the infrared radiation distribution and can be used to evaluate the differences in the external and internal structures of the sample. This is because the differences in the external and internal structures of the evaluated object will generate different heat conduction in the material, thereby affecting the heat flow [2]. This means that samples with defects, due to differences in internal structure, will cool or heat up at different ratios, resulting in different thermal contrasts in infrared thermal radiation imaging.

**Figure 1:**
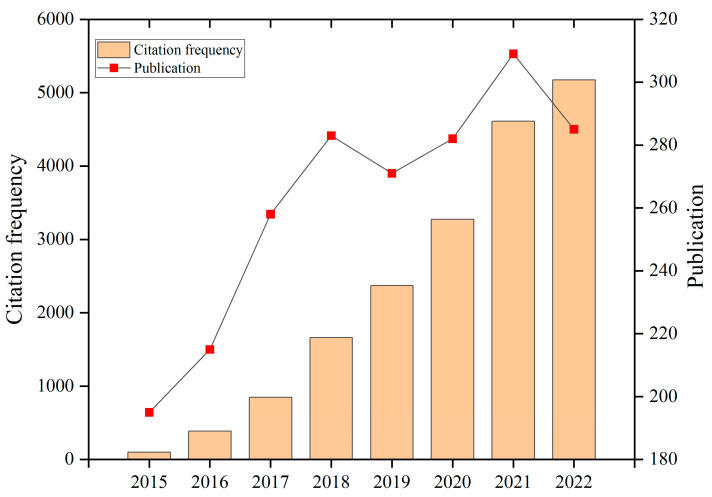
The citation frequency and number of publications for the keyword “IRT” were searched for in Web of Science.

Therefore, IRT technology can be used in the field of defect detection [3], especially in the electronic [4,5] and renewable industries [6]. According to the structural characteristics and defect properties of different materials, different types of thermal excitation sources need to be designed to actively heat the surface or interior of the tested object. The thermal excitation source can be modulated or not. Common excitation sources include flash/halogens lamps, hot air, lasers, ultrasound, electromagnetics, etc. Due to the presence of defects on the surface or inside of the tested object, there will be certain differences in the ways in which the thermal waves generated propagate towards the surface of the object. The main advantages of IRT over other technologies are: (1) non-contact and non-invasive; (2) high-speed; (3) large-area; (4) simple operation; (5) intuitive and easy-to-understand results; and (6) a wide range of inspection objects such as metallic, non-metallic, and composite materials. For example, with IRT technology, it is possible to measure the temperature of extremely hot objects or dangerous products (e.g., strong acid, hot steel) at high speed in a non-contact, non-invasive, and large-area way so that their temperature distribution can be safely measured and users can be kept away from danger [5,7]. In addition, it is possible to perform high-speed scanning not only of stationary targets but also of fast-moving targets. In contrast to the harmful radiation effects of techniques such as X-ray imaging, IRT is radiation-free and suitable for long-term and repeated use. Figure 1 illustrates the search results for the citation frequency and publication count of IRT keywords in the Web of Science database. The chart clearly reflects a gradual increase in both citation frequency and publication count, underscoring the continuous growth of research interest and study in the field of IRT. This upward trend suggests the increasing significance of IRT across various academic disciplines, motivating researchers to delve deeper into the applications and advancements of IRT technology. This is distinctly demonstrated in the chart, providing robust support and impetus for current and future IRT research.

In the past decade, the global photovoltaic (PV) market has grown almost exponentially in size. PV solar energy has strong competitiveness in the global energy market and has become a mainstream renewable energy technology [6]. The IRT imaging method is an efficient and potent tool for qualitative examination of PV modules when compared to conventional I–V characteristics. It can reliably pinpoint the specific position of defects in PV power plants in addition to detecting their presence in the system. For example, for a normal PV module, the incident irradiance causes a uniform temperature distribution on its surface. On the contrary, for most faulty PV modules, the thermal behavior of the PV module affects its surface temperature distribution, resulting in various inhomogeneities in the temperature distribution. This means that with minimal instrumentation, no direct contact, and no interruption of the functioning of the PV system in real-world conditions [8,9], details regarding the thermal characteristics and the precise physical location of the fault can be quickly obtained to quantitatively diagnose the presence of a faulty cell, cell bank, or module.

The electronics industry, stemming from the advancement and application of electronic science and technology, is not only one of the pillar industries of the national economy, but also an emerging science and technology development industry. In integrated circuits, for example, the electronic circuits of printed circuit boards (PCBs) are widely made [10,11], and these contain a high density of electronic components in the board power supply and many electronic connections, which are potential manufacturing defects. And the identification and localization of these defects are critical to the error-free performance of PCBs. Typically, defects produce abnormal temperature patterns that can be detected by the IRT. For example, transparent components are a key core component of smart terminals (one of the pillar industries of the electronics industry). Common transparent components mainly include the cover of the display, the light guide plate, etc., in this field [3]. 3D glass cover components are prone to defects (scratches, microcracks, microbubbles, water ripples, etc.) during the manufacturing process. According to statistics, the yield rate of 3D glass cover components is less than 75% [12,13,14], so high-performance detection of defects to improve the yield rate of the final smart terminal products is a technical challenge to be overcome. However, the defect detection process of transparent components has special characteristics with high light transmission and reflectivity, and the existing process is mainly manual. Relative to the traditional optical machine vision detection method, thermal spray infrared imaging will be controlled by a high-temperature gas through a moving nozzle, heating intelligent terminal transparent components due to the thermal resistance effect, component defects of the geometry, spatial location, etc. In the process of heat transfer, therefore, the defects in the vicinity of the spatial temperature evolution have a certain degree of variability compared to the normal, so the difference can be captured by thermal infrared imaging.

Due to the rapid expansion of the renewable energy sector, a dedicated section has been included in this paper to delve into the intricacies and developments within this industry. The fusion of IRT and advanced deep learning techniques represents a substantial leap forward in improving the accuracy and efficacy of detecting and diagnosing defects in PV panels [15]. For example, commonly used algorithms include convolutional neural networks (CNN), chaos synchronization detection method (CSDM), and genetic algorithm (GA) [16,17,18,19]. This integration harnesses the power of IRT’s thermal imaging to capture nuanced temperature variations across PV panel surfaces, and when combined with deep learning algorithms [20], the system can not only identify defects but also offer enhanced predictive capabilities [21]. Through this interdisciplinary approach, the ability to precisely pinpoint and diagnose issues in PV panels is substantially elevated [22,23].

The detection of electronic components presents a unique set of challenges owing to their complex and intricate structures [24]. In this context, the process of detecting defects and anomalies typically necessitates external excitation to induce heating within these electronic components. This, in turn, enables the capture of thermal radiation emitted by the object under inspection, facilitating the creation of a thermal image. In the realm of infrared thermal imaging for the detection of electronic components, lasers have emerged as a common and preferred excitation source [25,26]. The utilization of lasers at the 808 nm wavelength has demonstrated several advantages in electronic component inspection [27]. Firstly, it ensures the accurate targeting of specific areas of interest on the component, facilitating a controlled heating process. Additionally, this wavelength is well-matched to the spectral response of many infrared cameras, enhancing the efficiency of data acquisition [28]. As a result, the thermal images captured exhibit clarity and detail, enabling the detection of defects or anomalies with high precision.

## 2. Principle and Key Techniques

Active IRT is a technique whereby the surface or interior of an object to be inspected is excited in a controlled manner by a controlled heat source, causing its temperature to change. In this way, the changing temperature field of the object to be detected in space and time can be recorded using an infrared thermal camera to obtain the dynamic response of the heat wave. Afterwards, the thermal series of images obtained by the camera are processed and analyzed by image processing algorithms to determine whether the object to be detected is defective. In addition to high-performance infrared cameras, active IRT needs to focus on stimulating sources, heat transfer mechanisms, and image processing algorithms.

### 2.1. Principle

Active IRT, a subset of infrared imaging-based machine vision (IRMV), refers to a computer’s capability to produce images from infrared (IR) rays emitted or reflected by an object. A distinct demarcation exists between IRMV and traditional machine vision (MV). A typical IRMV system comprises an infrared camera with a lens, an infrared light source (stimulating heat source), a PC for image processing, a control module, and actuators. Traditional MV does not require the infrared camera and the stimulating heat source, but only a traditional camera and a common light source.

**Figure 2:**
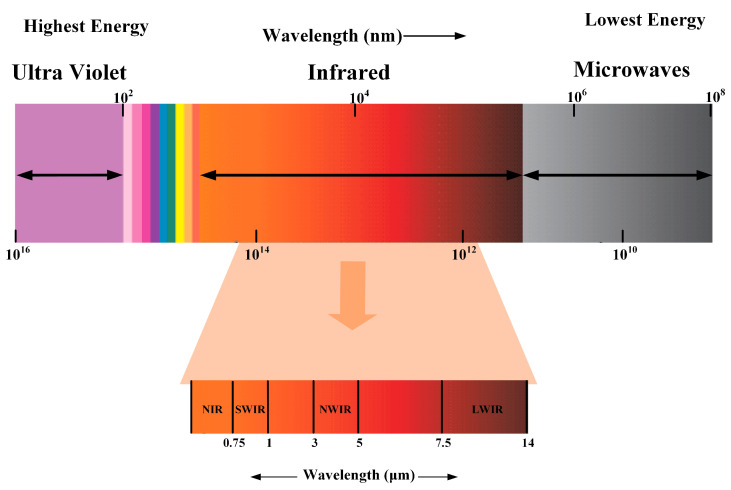
Infrared electromagnetic spectrum and its detection by infrared imaging.

Infrared is an electromagnetic wave with a wavelength between microwaves and visible light. The infrared band is usually subdivided into several sub-bands based on their wavelengths, as shown in Figure 2. Typically, near-infrared (NIR) waves have wavelengths ranging from 0.76 to 1 μm, short-wave infrared (SWIR) has wavelengths ranging from 1 to 2.5 μm, mid-wave infrared (MWIR) has wavelengths ranging from 3 to 5 μm, long-wave infrared (LWIR) rays have wavelengths ranging from 7.5 to 14 μm, and far-infrared (FIR) rays ranges from 15 to 1000 μm. Terahertz (THz) rays are FIR rays with wavelengths between 0.1 and 1 mm. As a result, TeraSense or another THz camera could be used to define terahertz machine vision (THzMV) [29]. Infrared applications are divided into three main categories: short-wave infrared, mid-wave infrared, and long-wave infrared. Short-wave infrared utilizes the short-wave infrared radiation prevalent in the target’s reflective environment and is similar in resolution and detail to visible light images. Long-wave and mid-wave infrared imaging utilizes thermal radiation emitted by the room-temperature target itself and is used in a variety of infrared thermal vision devices.

### 2.2. Excitation Sources

**Figure 3:**
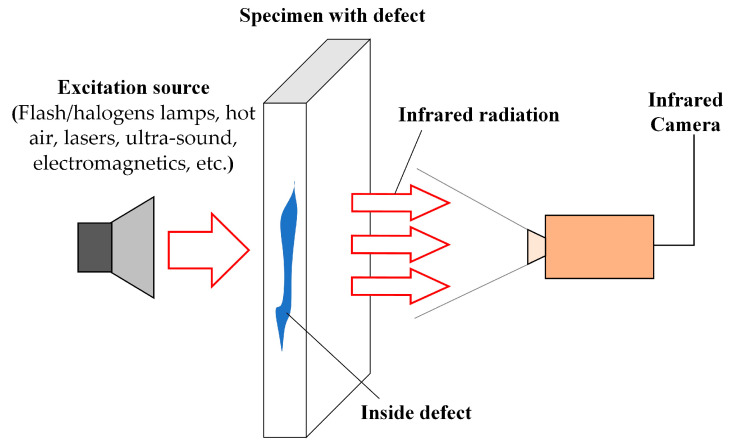
Active IRT method and its excitation source in the defect detection process.

Various excitation sources (e.g., flash/halogens lamps, hot air, lasers, ultra-sound, electromagnetics, etc.) are employed to thermally stimulate either the object’s surface or interior of the object according to the needs of different detection objects, to measure the temperature change after induction, as shown in Figure 3. Depending on the type of excitation heat source used, active IRT is mainly categorized into pulsed thermography (PT), locked-in thermography (LT), ultrasonically stimulated vibration thermography (UVT), and eddy current thermography (ECT).

**Figure 4:**
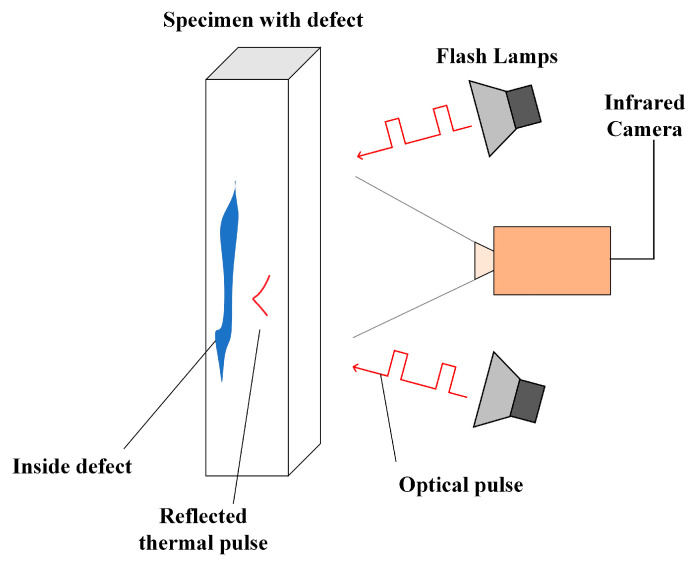
Schematic of PT tests in the defect detection process.

**PT** This technique utilizes a pulsed heat source (e.g., flash lamps, lasers, etc.) to emit heat pulses to the specimen into be inspected and heat it, as shown in Figure 4. Due to the very concentrated energy of the pulsed heat source and the very short pulses, the thermal equilibrium of the specimen is disturbed, and heat is rapidly conducted inside the specimen. If a defect exists, this results in a temperature difference between the surface of the specimen above the defect and the rest of the area. At this time, the fast infrared camera can record continuous thermal infrared imaging images, and the captured images are analyzed by the computer through algorithms for real-time pixel analysis. After the pulsed heat source is injected into the specimen, the variation of temperature profiles in different areas provides information about the internal defect characteristics of the material. This pulsed IRT can be used to detect defects on the surface or inside the specimen. The pulsed method has the advantage of being independent of compound heating inhomogeneities and possible changes in surface properties [4,30].

**Figure 5:**
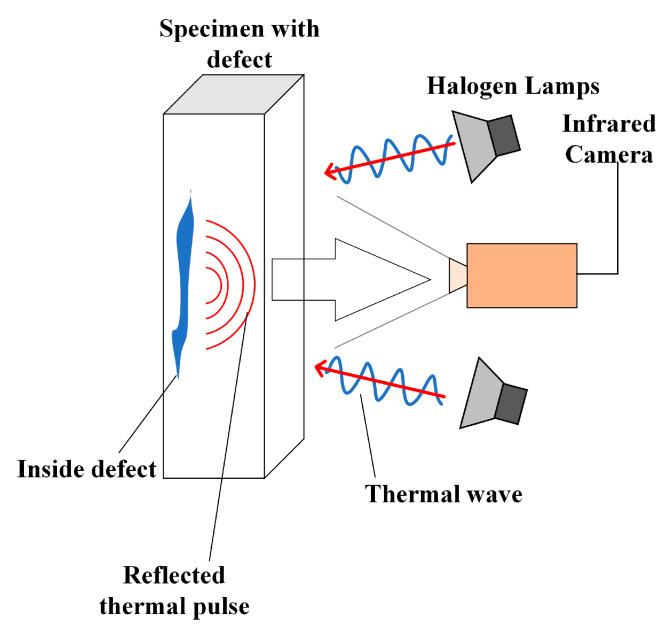
Schematic of LT tests in the defect detection process.

**LT** Lock-in thermography, also known as thermal wave imaging or modulated thermography, was proposed by Busse et al. [31], and has since been further developed by various researchers [32]. The method entails subjecting a specimen to a frequency-specified periodic (typically sinusoidal, with a given modulation frequency ω and amplitude I) thermal excitation in a steady state and then capturing the surface heating using an infrared camera. The thermal excitation time is at least one modulation cycle until the surface temperature of the specimen reaches a quiescent state. The detection method of LT is shown in Figure 5 and consists of a signal generator and an infrared camera. The signal generator provides the modulation frequency and intensity for the halogen lamp to generate thermal waves, and the IR camera has a high resolution to capture the thermal response of the specimen under thermal excitation. By analyzing the phase shift between the thermal excitation signal and the thermal surface response, it is possible to not only locate the presence of defects in the inspected specimen, but also to precisely locate the defects and determine the defect depth. The LT technique is used in much the same way as the PT technique, with the difference being the sampling frequency. In the former, the specimen to be tested is subjected to thermal excitation that lasts for several cycles, resulting in a longer testing time. However, this method is insensitive to external disturbances and works well even under difficult conditions. In addition, the LT technique allows defect detection on large surfaces, and the excitation frequency can determine the depth of test defects with a good signal-to-noise ratio.

**Figure 6:**
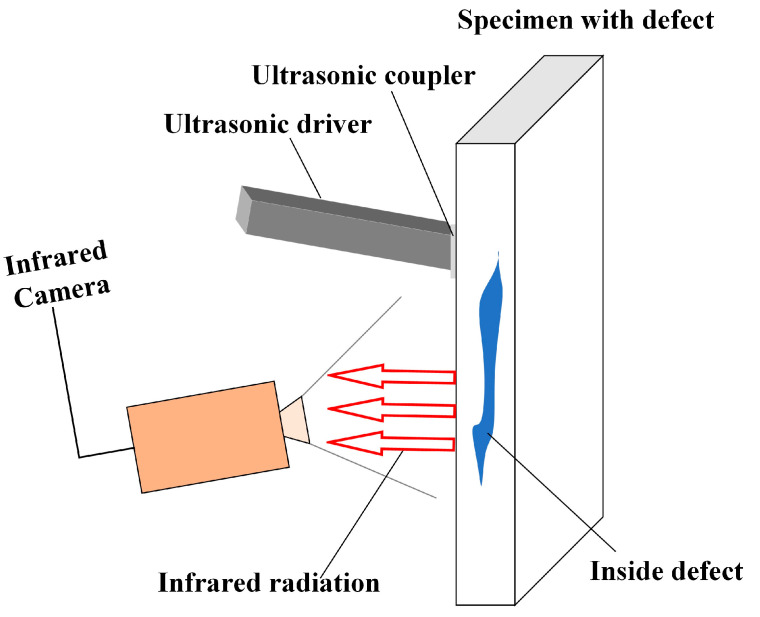
Schematic of UVT tests in in the defect detection process.

**UVT** It is well known that PT and LT are the two primary forms of optical excitation. Like optical excitation, acoustic excitation can also be used for active IRT detection. Figure 6 illustrates the schematic diagram of UVT, primarily consisting of an ultrasonic transducer and an infrared camera [33]. The ultrasonic transducer excites the specimen to be detected, and the vibration propagates inside the material, leading to localized heating of cracks through internal friction, and then through the high-performance infrared camera to capture its temperature changes. The interaction between the thermal and mechanical waves can localize the presence of defects in the specimen. If there are defects, because they release more heat through friction, there will be some difference in the thermal image from other normal areas. Since ultrasonic waves propagate to deeper layers, UVT can detect deep defects within the specimen. Analyzing the phase shift between the ultrasonic excitation and the thermal response enables precise defect localization.

**Figure 7:**
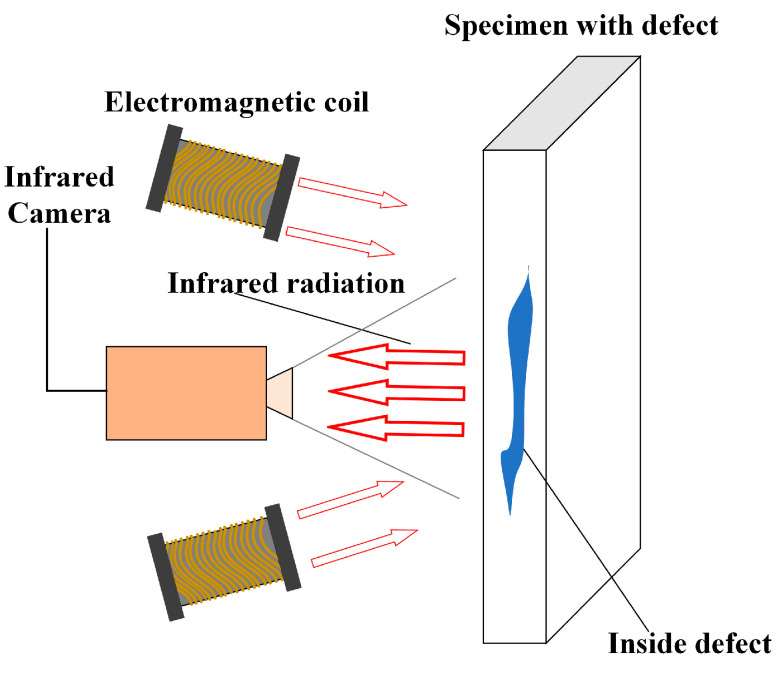
Schematic of ECT tests in in the defect detection process.

**ECT** Eddy-current-induced IRT is a technique that uses external excitation to induce eddy currents inside the specimen and an infrared camera to capture the heat flowing from the surface (shown in Figure 7) [34]. For instance, when a coil with pulsed excitation is brought near the test specimen, if the test specimen is free of defects and made of a uniform material, the induced eddy currents will be uniformly distributed across the test specimen. On the contrary, if the test piece surface or internal part has cracks and other defects or is mixed with other impurities due to material inhomogeneity, the induced eddy current will be around these defects or impurities, which will inevitably lead to the entire test specimen’s defective local temperature rising faster than the test specimen, thus forming a temperature field distribution.

### 2.3. Heat Transfer Mechanisms

In addition to the type of excitation heat source used for defect detection, the form of the excitation wave of the heat source is also very important due to the heat transfer mechanism involved. Evidently, manipulating the amplitude (energy level), frequency, and duration of the excitation heat source has a great impact on the outcomes of active thermography [35,36]. Suitable process parameters for the excitation heat source can enhance the accuracy and robustness of the detection. This means that for a specific specimen, we need to select the appropriate thermal excitation waveform according to the nature of the defect to better utilize the heat transfer effect and improve the signal-to-noise ratio between the defective region and the normal region. Thermal imaging techniques can be broadly categorized into two types: transient and static. The former is the use of pulsed (given the stimulus time of the waveform) forms of energy waveforms to stimulate the specimen to produce a thermal response; infrared data acquisition is carried out in the transient mode before the specimen is heated to a steady state. The latter is the use of modulated (given the frequency of the stimulus waveform) energy waves to stimulate the specimen to produce a thermal response. The specimen is heated to reach a steady state after infrared imaging to obtain the modulated waveform of the thermal response. Table 1 summarizes the energy waveforms and their equivalent temperature response. Take a point on the surface of the sample and a point on the defect, which is Sound P#1 and Defect P#2, respectively. The color of Sound P#1 is green, and the color of Defect P#2 is red. The colors of the curves correspond to the colors of the two points, respectively. The green curve shows what happens at the surface, while the red curve shows what happens at the defect site. The comparison of the two curves describes the different results produced by different methods in these two sampling areas, and the curve changes at the defect can be seen. The contrast between the two curves describes the different results produced by different methods in the two sample areas.

For transient heat transfer in the defect detection experiments, the material specimen is subjected to relatively short energy pulses and the temperature rise and decay curves over time are recorded. The diffusion of the thermal front under the surface of the specimen is calculated according to the Fourier diffusion equation [37], as shown in Equation (1):

(1) $$\frac{\partial T}{\partial t}=\alpha \nabla^{2}T$$

where α is the thermal diffusivity (m^2^/s).

**Table 1:** Summary of the energy waveforms and their equivalent temperature response for sound (P#1) and defective (P#2) points at the surface.

Specimen with Defect	Method		Heat Waveform	Temp. Response	Defect vs. Sound Response Schematic	Classic Theoretical Models
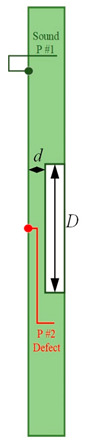	**PT**	**Dirac pulse** [38,39] (milliseconds, [40])	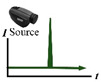	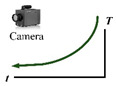	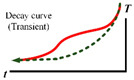	Refer to $T_{s}(z,t)=T_{0}+\frac{Q}{e\sqrt{\pi t}}\exp(\frac{-z^{2}}{4\alpha t})$ for 1D heat diffusion in finite body submitted to Dirac delta pulse.
		**Square pulse** [41,42] (seconds, [40])	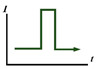	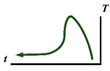	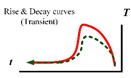	$\begin{array}{l} \mathrm{During}\;\mathrm{heating}:\;\\ T(0,t)=\frac{QL}{k}[F_{0}+\frac{1}{3}-\frac{1}{\pi^{2}}\sum_{n=1}^{\infty }\frac{1}{n^{2}}\exp(-n^{2}\pi^{2}F_{0})] \end{array}$ [41,42] $\begin{array}{l} \mathrm{After}\;\mathrm{heating}:\\ T(0,t)=\frac{QL}{k}[F_{oh}+\frac{2}{\pi^{2}}\sum_{n=1}^{\infty }\frac{1}{n^{2}}\exp(-n^{2}\pi^{2}F_{0})(\exp(-n^{2}\pi^{2}F_{oh}-1] \end{array}$$F_{o}=\alpha t/L^{2},F_{oh}=\alpha t_{h}/L^{2},\mathrm{where}\;t_{h}\;\mathrm{is}\;\mathrm{heating}\;\mathrm{time}$
		**Long pulse** [43] (up to 0.5 min, [40])	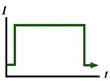	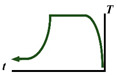	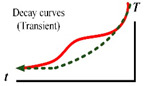	$\int_{0}^{t_{h}}T_{0}+\frac{Q}{e\sqrt{\pi t}}\exp(\frac{-z^{2}}{4\alpha t})dt$ [43]
	**Lock-in** [44]		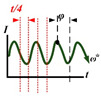	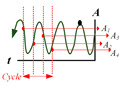	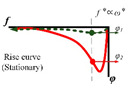	$T(0,t)=\frac{1}{2}T(0,\omega )(1+\cos\omega t)$ [45] $T(0,\omega )=\frac{Q}{k\sigma }[1+2\sum_{n=1}^{\infty }(-\Gamma^{n})\exp(-2n\sigma L)]$ $\varphi =\mathrm{Arctan}(\frac{A_{1}-A_{3}}{A_{2}-A_{4}}){|}_{\omega^{*}}$ $A=\sqrt{{\left(A_{1}-A_{3}\right)}^{2}+{\left(A_{2}-A_{4}\right)}^{2}}{|}_{\omega^{*}}$

$-\sigma =(1+)\sqrt{\omega /2\alpha }$: Complex thermal wave number; *k*: Thermal conductivity; *L*: Specimen thickness (it will be replaced with “*d*” for thickness over defect); *α*: Thermal diffusivity; *e*: Thermal effusivity.

Considering that the surface of the specimen is uniformly heated, the heat propagation into its interior can be regarded as a one-dimensional heat flow process [38,46]. Therefore, the one-dimensional heat flow of Equation (1) can be expressed as follows [37]:

(2) $$\frac{\partial T}{\partial t}=\alpha \frac{\partial^{2}T}{\partial z^{2}}$$

where *z* corresponds to the coordinate parallel to specimen thickness.

For a Dirac delta pulse plane source of strength *Q*/ρC released into a semi-infinite medium (*z* ≫ 0) from its surface (*z* = 0), Equation (2)’s solution is as follows [37]:

(3) $$T_{s}\left(z,t\right)=T_{0}+\frac{Q}{e\sqrt{\pi t}}\exp\left(\frac{-z^{2}}{4\alpha t}\right)$$

where $T_{s}$ is the transient temperature in the semi-infinite body, $T_{0}$ is the initial temperature, and *e* is the thermal effusivity.

**Static heat transfer** In the lock-in detection process, the specimen is subjected to periodic thermal waves and the one-dimensional solution for an isotropic semi-infinite specimen is as follows [47]:

(4) $$T_{s}\left(z,t\right)=T_{0}\exp\left(-\frac{z}{\mu }\right)\cos\left(\frac{2\pi z}{\lambda }-\omega t\right)$$

where ω is the modulated frequency, λ corresponds to thermal wavelength, $T_{0}$ is the initial temperature, and μ is expressed as thermal diffusion length, which is equivalent to the rate of decay of the thermal wave as it penetrates through the material [47].

In contrast to the PT technique (which records the temperature decay), the LT technique records the changes during the temperature rise period in a stationary state by means of a thermal imaging camera [35]. In the case of static heat transfer, the LT technique makes it easy to analyze the time dependence of the response waveform over a complete modulation period by using a sinusoidal waveform thermal excitation, which allows the reference waveform to maintain good shape and frequency, thus determining the type and location of defects.

### 2.4. IR Image Processing Algorithms

Compared to visible light imaging, infrared imaging characterizes the temperature distribution of the specimen and is a grayscale image with no color or shading, low resolution, and poor resolution potential. Therefore, the clarity of infrared imaging is lower than that of visible light images. Additionally, the infrared imaging process is susceptible to random external interference and imperfections in the thermal imaging system, resulting in a very low signal-to-noise ratio for the infrared image. This means that after IR imaging, when the acquired image does not provide satisfactory information about the condition of the detected object, it also needs to be preprocessed using appropriate algorithms [48,49]. Non-uniformity correction algorithms and image enhancement algorithms are typical representatives of IR image preprocessing algorithms.

It is well known that non-uniformity correction algorithms are mainly divided into two categories: calibration-based nonuniformity correction (CBNUC) and scene-based non-uniformity correction (SBNUC) [50]. CBNUC encompasses a range of algorithms used to mitigate non-uniformity in thermal infrared imaging devices. Representative algorithms in this category include two-point correction (TPC) [51], multi-point correction (MPC) [52], radiometric correction [53], and scene-based non-uniformity correction (SBNUC) [50]. Certain CBNUC methods can provide highly accurate non-uniformity correction, ensuring that thermal images accurately represent temperature differences. In some cases, the correction process may be time-consuming, especially when using SBNUC methods that require substantial computational effort [54,55]. As an example, the scene-based non-uniformity correction algorithm can adapt to the non-uniformity change caused by the ambient temperature change, in which the representative algorithms are the temporal high-pass filtering (THF) method [56], constant statistics (CS) method [57], Kalman filtering (KF) method [58], neural network (NN) method [59], and registration-based (RB) [60]. Infrared image enhancement algorithms mainly include traditional frequency domains, space domain, and new image enhancement methods. The traditional enhancement method is to adjust the histogram of the image through grayscale mapping so that its distribution is balanced to achieve the enhancement of the whole image contrast, which is fast and effective, suitable for the scene depth, and does not change much. At the same time, the image distribution is relatively uniform. Most of the traditional algorithms are based on the histogram equalization (HE) algorithm for infrared image enhancement, which can be classified into two categories according to the area of action of the mapping function: the global contrast enhancement (GCE) algorithm and the local contrast enhancement (LCE) algorithm [61,62]. Among the new image enhancement methods, Edwin Land proposed the Retinex theory, an image enhancement algorithm that removes the effect of irradiated light in the original image and obtains the reflective properties possessed by the object itself [63] to analyze the intrinsic nature of the image. This algorithm has the advantages of local contrast enhancement, high dynamic range compression, and image color constancy that can be maintained.

Recently, with the development of MV and artificial intelligence (AI) image processing techniques, the level of a computer’s ability to process and comprehend images has increased [64]. Machine learning (ML) is a branch of AI that learns from data through computer programs and automatically improves and adapts its performance [65,66]. Thus, ML aims to help computers learn and adapt, without having to perform explicitly extensive manual programming, to automated analytical algorithms that deal with multivariate and multiparameter problems [3]. For example, Saintey and Almond [67] utilized an artificial neural network (ANN) as an expert system to obtain detailed information on defect size and depth from transient thermographic data. This type of method [67,68] is generally based on pre-training the ANN on normal, defect-characterized experimental datasets to obtain the thermal contrast, phase contrast, etc., after infrared imaging as a function of the presence or absence of defects, the defect shape and size categories, and the range of defect depths. Notably, clustering algorithms (including various improved versions) have also been widely used in defect detection in IR imaging [8,69,70].

## 3. Renewable Industry

PV solar power generation has become an indispensable component of the global energy landscape [71,72]. The long-term performance and overall reliability of PV modules are significantly influenced by faults occurring both in real-world operational conditions and during transportation and installation [73,74]. These faults lead to specific abnormal operations, primarily characterized by reduced power output, abnormal module surface temperature distribution, excessive thermal/mechanical stress, and even safety risks [75,76]. Traditional electrical performance testing of PV modules is a mature testing method, but it has limited fault-detection capabilities [77]. With the advent of digital cameras, charge-coupled devices (CCDs), and uncooled focal plane array (UFPA) detectors, optical-based infrared thermal imaging detection has gained popularity [78,79]. Specifically, electroluminescence (EL) and IR imaging prove to be potent tools for the qualitative assessment of PV modules, enabling the detection of faults in PV installations and precise identification of their exact locations [80]. In conducting this research, a total of 94 literature reviews published between 2000 and 2023 were identified on the Web of Science. These reviews covered various domains, including energy fuels, engineering, and computer science, and were obtained by limiting the search to reviews related to IRT detection in PV. Table 2 presents the top five most-cited reviews in this domain.

Regarding the number of literature searches on the infrared detection of photovoltaic panels in Web of Science, Table 3 provides an overview of key annual performance indicators. The table demonstrates a noticeable upward trajectory in the volume of the literature, reflecting the growing interest in the research field. However, it is worth noting that both the average citation count and H-Index for each publication exhibit a declining trend, as indicated in Table 3. This trend can be attributed to the tendency for older literature to accumulate more citations. Of particular interest is the anomaly in 2019, where, despite a substantial increase in publications, there was a sharp decrease in the average citation count per publication and the average yearly citation rate per publication.

**Figure 8:**
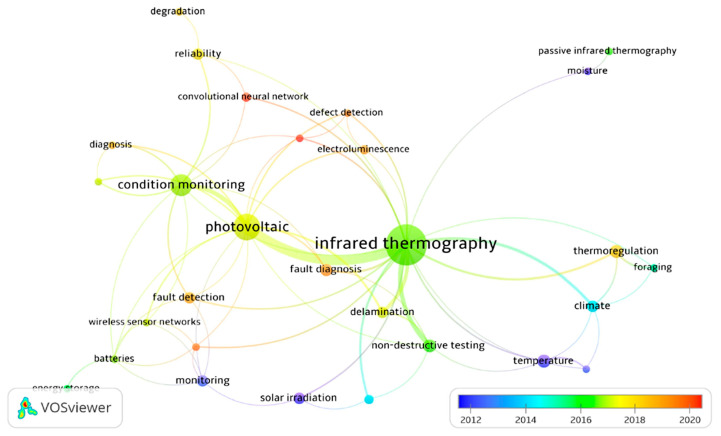
Map of keywords co-occurrence in IRT-PV context [21].

Numerous investigations have been carried out, and there has been a recent surge in publications focusing on assessing the suitability of IRT for the detection of PV anomalies [84]. Kandeal et al. [21] accomplished this by meticulously analyzing the available data from the Scopus database and using the VOSviewer tool [85] to create a bibliometric network to illustrate the literature. These networks were presented in the schematic representation of keyword relationships (Figure 8). In this illustration, the size of each circle signifies the occurrence frequency of keywords, the thickness of the connecting lines represents how frequently these keywords co-occur, and the color-coding denotes the year of publication. As depicted, the IRT method has been widely employed across various imaging applications and has found substantial utility in the monitoring of PV conditions, particularly from 2016 onward.

### 3.1. Optical Degradation

**Figure 9:**
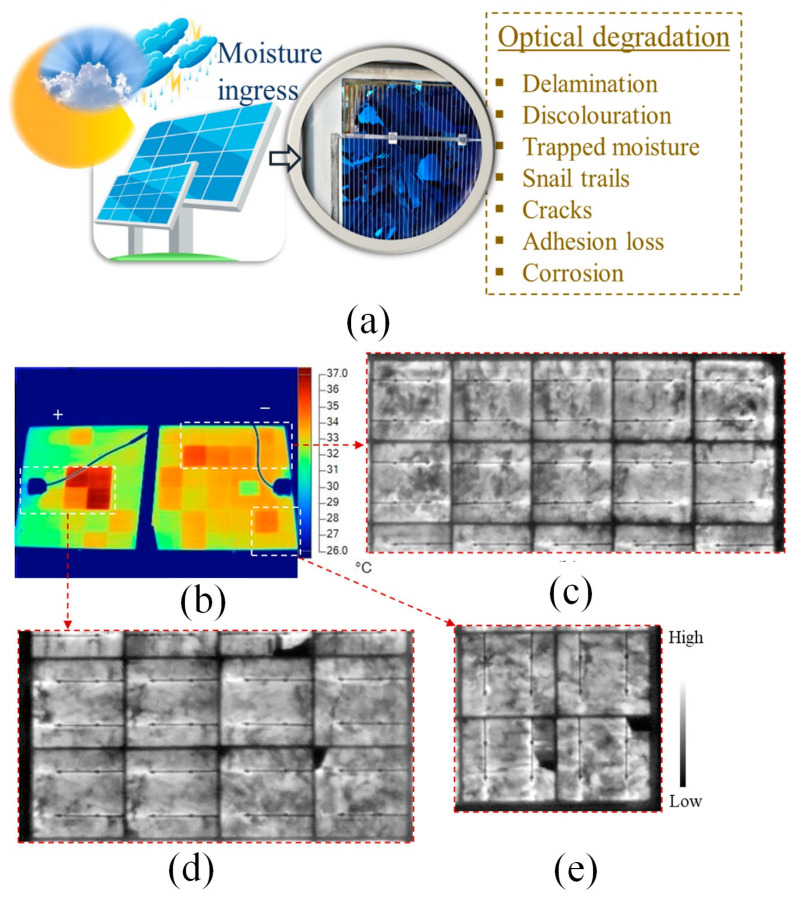
PV module in the field: (**a**) under environmental stressors e.g., high humidity, temperature, and UV radiation, moisture can enter the PV module [92]; (**b**) IRT characteristics of PV Module X acquired under clear sky outdoor conditions; (**c**–**e**) EL characteristics acquired under *I_sc_* bias conditions of the corresponding marked areas in (**b**) [97].

One of the pivotal attributes of high-quality PV front encapsulation materials is achieving optimal optical transmission efficiency [86,87]. However, when deployed in real-world conditions, PV modules encounter an array of environmental challenges, including elevated temperatures, humidity, exposure to ultraviolet (UV) radiation, wind, and snow pressure [88,89]. Among these environmental stressors, moisture can infiltrate the interior of the solar panel through various pathways, including its edges, rear section, or any voids like cracks in the panel structure [90,91]. The pathway leading to optical deterioration in PV modules as a consequence of moisture infiltration is depicted in Figure 9a [92]. As time progresses, the concern regarding optical degradation intensifies and, in the most severe scenarios, can result in a reduction of over 50% in the rated power output of the PV module [93]. Therefore, it is crucial to understand the attributes of imperfections and the fault mechanisms responsible for the optical deterioration of PV devices. This understanding is essential for preventing further degradation and the development of additional failure mechanisms [94].

IR imaging offers insights into the temperature distribution across the surface of the PV module and the location of defects or fault modes [95]. Faulty cells result in mismatch losses, thereby leading to an uneven distribution of cell temperature (*Tc*) across the PV module. The malfunctioning cells operate at elevated *Tc* levels, creating hotspots that subsequently affect the module temperature (*Tm*) [96]. Figure 9b displays the IR image of PV Module X, while Figure 9c–e present magnified EL images of the highlighted regions in Figure 9b [97]. These highlighted areas in Figure 9b are in proximity to the module’s frame and represent the most critical hotspots, indicating the presence of significant leakage current during operation. It’s worth noting that hotspots are distributed throughout the module. The positioning of hotspot cells near the PV module’s frame aligns with the findings from the electroluminescence (EL) images. The abundance of hotspot cells implies that a substantial portion of the cells in field-aged PV Module X are experiencing various stages of degradation. In Figure 9c, no evident cracks are detected, but the highlighted region in Figure 9b shows hotspots. These hotspots in Figure 9b may result from metal grid corrosion and/or solar cell degradation. Moving to Figure 9d, it reveals the existence of microcracks, with the warmest cells identified in this area on the IR image (as seen in Figure 9b). In contrast, Figure 9e displays some cracks, but the hotspots in its corresponding area on the IRT image are not as pronounced as those in Figure 9d. The significance of cracks in facilitating current flow underscores the occurrence and severity of the hotspots observed in Figure 9d. The Δ*T* of PV Module X was approximately ∼8.2 ± 2 °C [98].

Addressing the current drawbacks in industrial production lines, such as low defect detection efficiency, limited data, and high error rates, is crucial due to the significant impact of defects in the silicon photovoltaic (Si-PV) cell manufacturing process on the normal power generation of PV systems. Hence, defect detection is of utmost importance. Du et al. [99] introduced a defect detection and classification method for Si-PV cells based on IRT and CNN. The method involved fine-tuning the LeNet-5, VGG-16, and GoogleNet models after generating the dataset. After 71 training iterations, the GoogleNet model consistently achieved 100% defect classification accuracy with a verification accuracy of 100% and a loss of 0.002. However, training was halted at this point since no significant improvements were observed, and the model reached its peak stability at the highest accuracy. The VGG-16 model attained its highest defect classification accuracy after 121 training iterations, achieving a verification accuracy of 97.67% and a loss of 0.15. While the LeNet-5 model could also achieve a 100% precision value, it exhibited instability and significant fluctuations during the training process. Balasubramani et al. [100] proposed a method for detecting ethylene vinyl acetate (EVA) discoloration and delamination defects based on the thermal pixel counting (TPC) algorithm. Temperature indicators, namely T_15_ and T_20_, were introduced to highlight the temperature pixel distribution at ΔT °C = 15 °C and 20 °C, respectively. These indicators were compared with healthy panels to validate the algorithm’s effectiveness. The classification was automated using a fuzzy classifier, adjusting classification boundaries by modifying fuzzy IF-THEN rule certainty levels while keeping membership function parameter values constant. This approach, particularly the use of the certainty factor (CF) in the fuzzy classifier, significantly improved classification accuracy, surpassing other methods by an average of 10%.

### 3.2. Electrical Mismatches and Degradation

The term “electrical mismatches” encompasses a range of fault types, including cell cracks, snail trails, broken interconnecting ribbons and busbars, shunts, and poor soldering [100]. These faults are not always discernible through straightforward visual inspection, especially when it comes to optical degradation. Typically, power loss and thermal degradation in faulty modules can lead to an increased risk of safety issues in the entire PV system [6]. Mismatched voltage characteristics can lead to uneven current distribution, thereby affecting the overall performance of the system. Current imbalances between different components may result in electrical mismatch issues among the modules [101].

**Figure 10:**
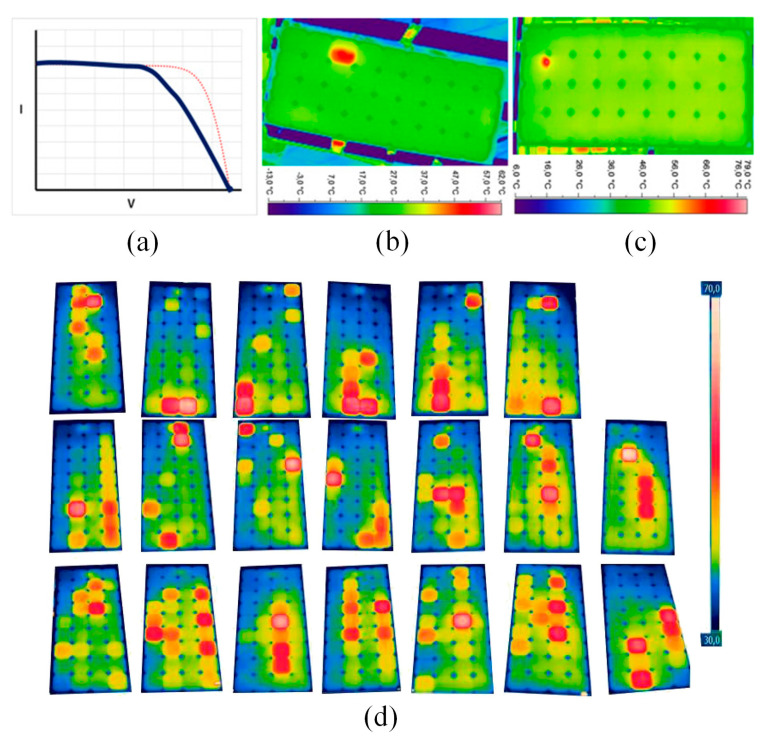
PV module in the field: (**a**) typical *I*–*V* characteristic output [6]. IRT of an electrically mismatched PV module, due to broken interconnection ribbons; (**b**) defective soldering/busbar (**c**) [6]; (**d**) thermal image of PV module [105].

The commonly employed method for diagnosing faults in solar PV panels is the measurement of Current–Voltage (*I*–*V*) characteristics. However, this approach is time-consuming and lacks the ability to categorize defects like delamination, discoloration of EVA, and isolation of cell parts resulting from cell cracks [102]. Pei and Hao [103] presented fault indicators based on current and voltage to detect faults in PV systems. According to the experimental results by Tsanakas et al. [6], cracks in PV modules were actually diagnosed through the *I*–*V* characteristics. These interconnection material issues in a single cell or within a cell string occur due to physical strains during transport or installation, thermal cycling leading to thermomechanical stresses, subpar soldering, and potential hotspots arising from extended PV system operation in real-world conditions [104]. Detecting broken interconnections is straightforward using optical techniques such as EL, IRT, ultraviolet (UV) imaging, or through basic *I*–*V* characterization (see Figure 10). Figure 10a illustrates the typical *I*–*V* characteristic output. Figure 10b,c display the thermal images of PV modules with electrical mismatches, attributed to interconnection ribbon fractures (Figure 10b) and soldering/busbar defects (Figure 10c) as observed through IRT. Figure 10a shows the typical *I*–*V* characteristic output, while Figure 10b,c display thermal images of PV modules. These thermal images reveal electrical mismatches due to interconnection ribbon fractures (Figure 10b) and soldering/busbar defects (Figure 10c), as observed through IRT. Belhaouas et al. [105] employed thermal imaging to investigate the performance of solar PV modules after outdoor exposure. The thermographic inspection revealed that the temperature of PV cells inside the PV modules ranges from 32 °C to 68.2 °C, as given in Figure 10d. This temperature variation occurs while the average ambient temperature during the thermal inspection is 23 °C. The thermal inspection found that the deployed PV modules, regardless of their glass types, primarily experience minor temperature mismatch (Δ*T*) at 90.27%, followed by major Δ*T* mismatch at 9.58%, and a critical Δ*T* mismatch case at 0.13%. Nonetheless, PV modules with textured glass exhibit slightly lower thermal stress levels compared to those with float glass. Tsanakas et al. [106] assessed the suitability of thermal image processing and edge detection for defect detection in PV modules. The approach combined image segmentation with Canny edge detection and has yielded favorable results through on-site thermal imaging measurements of two PV arrays: PV-1 and PV-2. It successfully identified 13 out of 14 faulty cells in PV-1 and 27 out of 29 faulty cells in PV-2 by detecting hotspots within the edge maps. These identified hotspots were validated against the standard electrical tests conducted on each module before the experiments, revealing a performance decline of 9.5% for PV-1 and 9.7% for PV-2, respectively. Aziz et al. [107] exploited continuous wavelet transform to generate two-dimensional (2D) images from PV system data and utilized CNN for PV system fault classification, achieving a circuit fault detection accuracy of 73.53%.

### 3.3. Non-Classified Faults

In addition to optical degradation and electrical mismatches and degradation, faults such as potential-induced degradation (PID) and defective bypass diodes (short circuits) are informally referred to as “non-classified” faults. PID is a relatively newly identified fault mechanism in operational PV modules and remains an area with limited comprehensive research and understanding. It involves a crucial externally induced factor, typically accelerated in hot and humid conditions, resulting in significant degradation and power loss within the affected PV modules [108,109].

**Figure 11:**
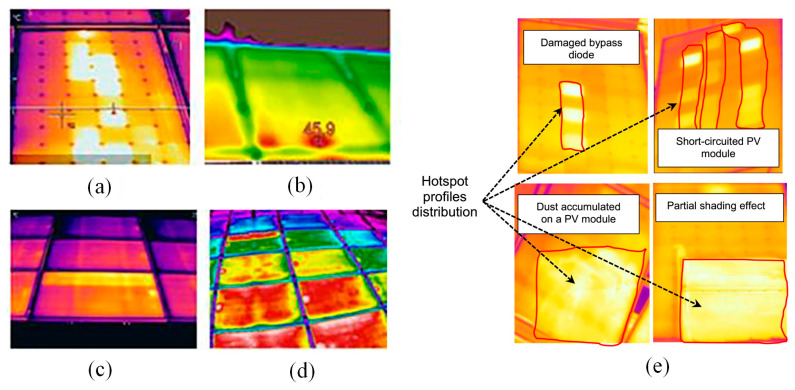
Different thermographic images used: (**a**) thermal images taken by operators using standard lenses [18]; (**b**) the thermographic image was captured at an angle that is not perpendicular to the module [18]; (**c**) thermal image obtained by standard lens [18]; (**d**) thermal image obtained by wide-angle lens [18]; (**e**) host spot profiles variation for different examined PV module defects [111].

Researchers have conducted various algorithm and laboratory tests to detect “non-classified” faults. For instance, Bouaichi et al. [110] assessed the PID recovery process in affected PV modules using IR evaluation. PID can be considered a factor affecting the durability and power output of crystalline silicon modules. Lu et al. [16] employed a hybrid algorithm combining chaos synchronization detection method (CSDM) and CNN for the investigation of fault detection in PV modules. The discussion encompassed four prevalent states observed in PV modules: the normal state, module damage state, module contact defect state, and module bypass diode failure state. The research findings showcased the proposed method’s remarkable recognition accuracy of 99.5% when 400 sets of randomly generated fault data (with 100 data points for each fault) were inputted, surpassing the traditional edited nearest neighbor (ENN) algorithm’s recognition rate of 86.75%. Tao et al. [17] introduced a genetic algorithm-optimized deep belief network (GA-DBN) for diagnosing PV faults, covering normal operation, grounded short circuit, open-circuit in series, partial shadow, and abnormal aging. Although achieving an impressive overall diagnostic accuracy of 95.73%, it’s important to note that the average training time was relatively long at 316.34 s, primarily due to the intricate optimization process involving the initial weight and bias of the DBN through GA. Manno et al. [18] achieved optimal performance with CNN using thresholding as a preprocessing method, achieving a 99% accuracy on mid-range CPUs in less than 30 min. Additionally, simplification of thermal imaging images, representing various operational states of PV modules, can achieve high precision. Considering a dataset consisting of 200 sliced images, the same configuration resulted in 90% accuracy for the MLP network and 100% accuracy for CNN. Figure 11 displays various thermographic images utilized for CNN training, the thermographic image in Figure 11a was taken by an operator using a standard lens. Figure 11b shows a non-perpendicular thermographic image angle, and Figure 11c, captured with a standard lens, includes multiple PV modules. In Figure 11d, the thermographic image was acquired using a wide-angle lens and encompasses several PV modules. Mellit [111] adopted an embedded system for fault detection and diagnosis in PV modules, utilizing IRT and deep convolutional neural networks (DCNNs). Two DCNN-based models were developed, one for fault detection and the other for fault diagnosis. Despite the limited dataset size, simulation results indicate a remarkable accuracy of 99% for fault detection and a quite impressive 95.55% accuracy for fault diagnosis. As shown in Figure 11e, the classifier accurately identifies instances of dust deposition on the PV surface, with a recognition accuracy of only 95.5%. In fact, this is due to the similarity in contours between partial shading effects and dust accumulation, as well as PV modules with short circuits and damaged bypass diodes. Dhimish et al. [112] imported a novel PV hotspot fault detection algorithm based on cumulative density function (CDF) modeling technique, achieving an accuracy of 80%.

### 3.4. Summary

The advantages of the machine-learning-based method over traditional methods are manifold. Machine learning algorithms can adapt and learn from data, allowing them to improve their performance over time without the need for manual adjustments. This adaptability is a significant advantage when dealing with complex and dynamic systems [113]. Machine-learning-based methods undoubtedly offer numerous advantages for IRT applications. However, like any approach, they do come with certain disadvantages that need to be considered in the context of IRT. Machine learning models, especially deep learning models, require large amounts of data for effective training. In the case of IRT, acquiring a substantial dataset, particularly for rare or specific defects, can be challenging [114]. Furthermore, efforts must be made to make machine learning models more interpretable and transparent in the context of IRT to establish trust and confidence in their results.

Machine learning is a widely-used technology that relies on algorithms and models to enable computers to learn from data and make decisions. Deep learning, on the other hand, is a branch of machine learning that involves artificial neural networks, which can simulate the workings of the human brain to process vast amounts of complex data. The integration of IRT with deep learning plays a pivotal role in detecting and diagnosing defects in PV panels [115,116]. Initially, the technique of IRT is employed to capture thermal images of the PV panels. These thermal images depict the temperature distribution across the surface of the PV panels, where defects typically manifest as anomalous temperature patterns. Preprocessing of the thermal images may be necessary to eliminate noise, enhance contrast, or adjust image dimensions to ensure compatibility with deep learning models [117]. Deep learning models [118], such as CNN [99] or GA-DBN [17], are then utilized to learn and extract features pertaining to defects from the thermal images [119]. These models possess the capability to autonomously acquire knowledge and recognize patterns within the thermal images, including potential defects. The deep learning models excel in automatically discerning complex patterns and temperature distributions within the IRT, thereby enhancing the accuracy of fault detection and diagnosis [120]. This amalgamation enables the automation of the detection and diagnosis processes, reducing the reliance on manual intervention and significantly enhancing overall efficiency.

**Figure 12:**
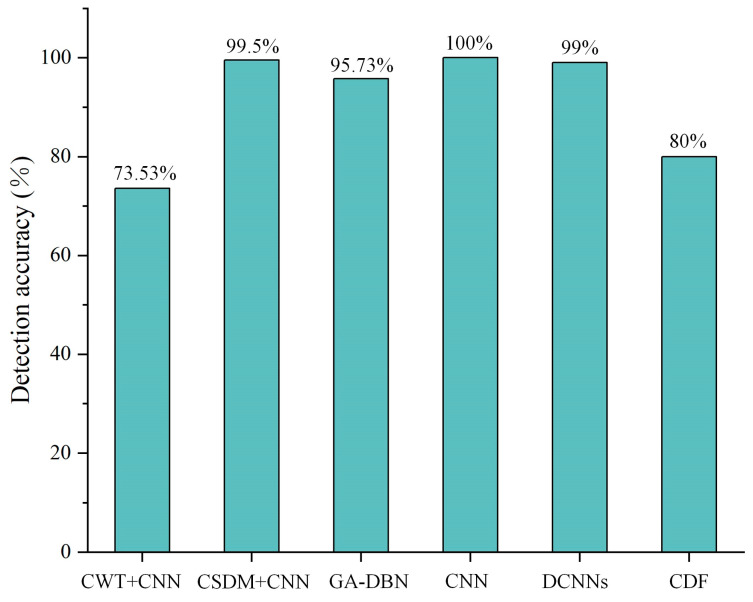
PV panel defect detection accuracy.

For instance, despite the relatively limited scale of the dataset employed in Mellit’s study [111], simulation results demonstrated a fault detection accuracy of 99% and a fault diagnosis accuracy of 95.55%, as shown in the Figure 12. In most cases, this method can identify various types of defects in PV panels, including but not limited to hotspots, cracks, dirt, and cell damage. Performance metrics for detection may encompass accuracy, recall, and precision, among others, and these metrics are generally contingent on the specific problem and model configurations. In summary, the fusion of IRT and deep learning offers an efficient and highly accurate solution for detecting defects in PV panels. It holds the potential to play a crucial role in the monitoring and maintenance of PV energy systems. Table 4 summarizes the application of the combination of IRT and deep learning techniques for defect detection and diagnosis of PV panels.

Table 5 presents a comprehensive comparative analysis between research conducted by scholars in the past and the current state of research. Historically, the majority of studies were primarily focused on the conventional methods for PV panel inspection. In contrast, contemporary scholars are placing significant emphasis on the integration of deep learning with IRT techniques. This shift in focus reflects the evolving landscape of research in this field and the recognition of the potential of advanced methods for more precise and efficient PV panel defect detection. The utilization of deep learning in conjunction with IRT is emerging as a promising avenue for achieving higher accuracy and reliability in the inspection of PV panels.

Automatic photovoltaic inspection has garnered significant interest from researchers in recent years. Numerous studies have explored automatic photovoltaic inspection using various imaging methods. Demant et al. [130] employed a support vector machine algorithm for the automatic classification of cracks in photoluminescence (PL) images. Stromer et al. [131] proposed an enhanced EL image crack segmentation framework. Li et al. [132] adopted image processing algorithms for the automatic detection of snail trails and dust in visible light images. Su et al. [133] utilized newly proposed feature descriptors to classify manufacturing defects in solar cell EL images. However, there has been limited research on the application of deep learning for defect detection in photovoltaic component images. These studies, including those by Chen et al. [134], Ding et al. [135], and Li et al. [136], have leveraged deep learning techniques to detect defects in visible light (red, green, blue) RGB images. Demant et al. [137] used CNN for automatic quality assessment and control during the production of solar cells in PL images. Deitsch et al. [138] and Akram et al. [139] employed deep learning methods for the automatic detection of faults in solar cell EL images. This represents a notable shift toward utilizing deep learning approaches for photovoltaic inspection.

## 4. Electronic Industry

With the progression of information electronic devices towards high reliability, miniaturization, light weight, and multifunctionality, high-density integrated circuits with numerous functional components have found extensive applications [140,141]. PCBs, serving as critical structures for electrical and pneumatic interconnection, signal transmission, mechanical linkage, and electronic system support, are also the primary failure-prone areas of components. The long-term reliability of PCBs has become a focal research topic, resulting in challenges associated with effectively and reliably detecting PCBs’ defects. Traditional PCB defect detection methods have limitations, but active IRT, including techniques like pulsed thermography and lock-in thermography, has found extensive use in non-destructive testing for PCBs. The development of very large-scale integration (VLSI) technology, increasing silicon wafer diameters, and decreasing integrated circuit linewidths have imposed higher demands on silicon wafer manufacturing processes and surface quality [142]. During semiconductor silicon wafer production, the formation of microcrack defects is common, ultimately affecting the quality of silicon-based microelectronic products. Ensuring the quality and performance of products necessitates non-destructive testing of silicon wafers. Surface mount components achieve interconnection between chips/packages and substrates or PCBs using solder bumps. However, common manufacturing defects, including opens, cracks, or missing solder bumps, persist. As solder bumps are concealed within packages after assembly, the increasing trend towards high-density and ultra-fine pitch has made defect detection progressively more challenging, severely impeding the advancement of surface mount technology. Detecting defects in solder bump protrusions has become a critical issue in integrated circuit manufacturing technology. Concealed solder bump protrusions impede the entry of light beams, and infrared imaging proves to be an effective detection technique capable of identifying nearly all solder bump defects.

Refer to the number of literature searches on Web of Science on the application of infrared thermal imaging technology in electronic industry defect detection, and the results are shown in Table 6. Table 6 provides an overview of the key annual performance indicators. As can be seen from the table, the number of literatures is in a slightly fluctuating state each year, indicating that people’s interest in this field has not changed much. It is worth noting that the average number of citations and the H-Index of each publication are almost horizontal, but suddenly decline in 2022. This trend can be attributed to the lack of in-depth research in the field. Of particular interest is the anomaly of 2017, in which the average number of citations per publication rose sharply, even though the number of publications was not as high as before.

Table 7 makes a comprehensive comparative analysis of the research of past scholars and the current research status. Historically, the feasibility of infrared non-destructive testing technology has brought a lot of convenience to the electronics industry, and accumulated experience for the subsequent research. With the improvement of technology and the deepening of research, it can be seen that contemporary scholars have added the cost factor to the concern of non-destructive testing in the electronics industry. Future cost reductions will also make infrared non-destructive testing technology have a better market.

### 4.1. Chip

Since the 1960s, advancements in semiconductor technology have profoundly transformed our lives and facilitated the development of high-performance electronic devices. The emergence of smartphones, for instance, would not have been possible without the progress in miniaturized and high-performance semiconductors. The demand for lighter, more compact smartphones necessitates the production of smaller, thinner, and higher-performing semiconductor chips. With the growing trend of using thinner wafers for semiconductor chips, various issues have emerged, including a significant concern related to microcracks that can be found on the surface and sub-surface, varying in size from a few micrometers to several tens of micrometers. Semiconductor chip materials are inherently brittle, making them susceptible to stress-induced cracks during chip manufacturing and assembly. These cracks manifest primarily as scratches, fractures, orange peel effects, and pits [150]. Surface cracks can adversely affect the performance and reliability of the final electronic device, thus escalating the demand for inspecting surface cracks in semiconductor chips during the manufacturing process. Efficient and high-precision non-destructive testing is crucial for semiconductor chip inspection. Optical visual methods, while offering non-contact and non-destructive three-dimensional chip characterization, have limitations in detecting concealed defects. Active IRT bestows the following advantages for semiconductor chip inspection: complete non-contact, non-destructive, and non-invasive testing, along with the capability to examine large areas in a single test. IRT has emerged as one of the most promising techniques in non-destructive testing and evaluation [145].

**Figure 13:**
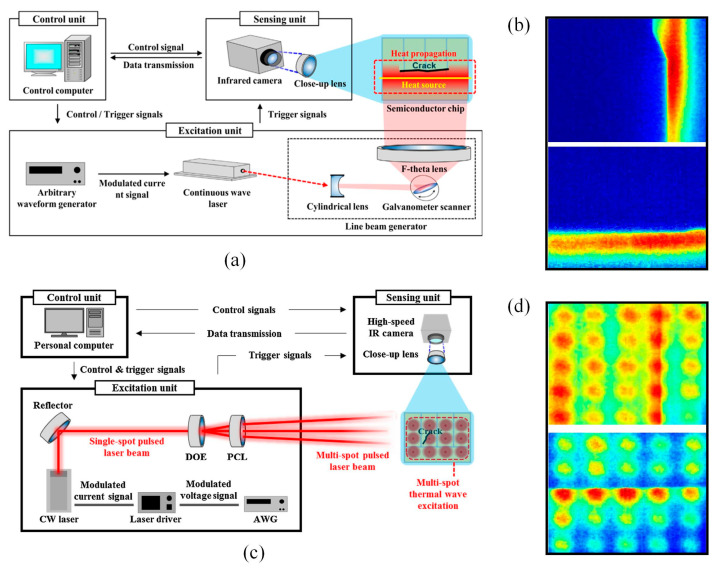
Schematic representations of two distinct laser-based thermographic inspection methods: (**a**) line laser lock-in thermography system and (**b**) corresponding thermograms of vertically oriented crack chips and horizontally oriented crack chips within this system [26]; (**c**) multi-point laser lock-in thermography system and (**d**) corresponding thermograms of vertically oriented crack chips and horizontally oriented crack chips within this system [152].

Introducing non-contact active IRT technology into chip defect detection involves the use of an external heat source, such as a flash lamp or laser, for active thermal imaging. When subjected to external heating, the presence of defects within the chip leads to abnormal thermal resistance, enabling the capture of thermal distributions using an infrared imaging device. Analyzing thermal images aids in defect identification, with laser excitation being the most frequently used method for semiconductor chip defect detection among various external excitation techniques. Bu et al. [151] investigated a method utilizing Barker code-modulated pulse compression waveforms for detecting microcrack defects in semiconductor silicon wafers. This technique employed an optical infrared thermal imaging device for transmission, where an infrared camera captured the thermal wave signal response to the laser-modulated Barker code waveform. The acquired images were stored as sequences and analyzed for detectability using a full-harmonic distortion algorithm, resulting in improved defect detectability. An et al. [26] introduced the line laser lock-in thermal imaging technique for semiconductor chip inspection. This technique integrated a line-scanning laser source, an infrared camera with a dedicated lens, and a control computer, assembling a novel line laser lock-in thermal imaging system as shown in Figure 13a. The continuous wave laser beam was modulated into a pulsed laser beam by the excitation unit, and the cylindrical lens transformed the pulsed laser beam shape from point-like to linear. The control unit then issued control signals to the galvo scanner, directing the line laser beam onto the target surface. Subsequently, the line laser beam generated a thermal wave along the desired excitation line, performing horizontal and vertical scans on the target surface, effectively detecting randomly oriented cracks, as shown in Figure 13b. Yang et al. [152] proposed a multi-point laser lock-in thermal imaging system for real-time imaging of semiconductor chip cracks, as shown in Figure 13c. This system employed multi-point pulsed laser beams to simultaneously generate thermal waves at multiple points on the target semiconductor chip surface. The corresponding thermal response was measured using a high-speed infrared camera, enabling real-time detection during the semiconductor chip manufacturing process. Figure 13b,d illustrates a comparative diagram of semiconductor chip defect detection using the same excitation source—laser—in different modes. The integration of infrared sensing technology with the lock-in method significantly improved the sensitivity and resolution of thermal imaging. The sensitivity of thermal imaging was increased by two orders of magnitude, reaching approximately 100 μK, while the resolution for surface defects was lowered to 5 μm [153].

### 4.2. PCBs

**Figure 14:**
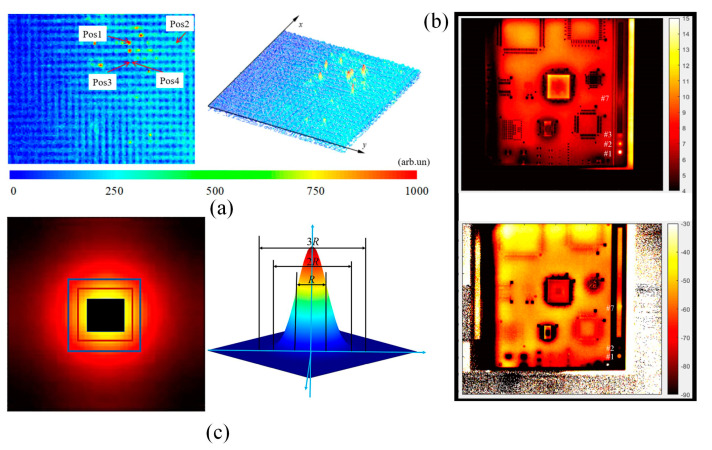
IRT in PCBs: (**a**) 2D and 3D views of transient amplitude images [158]; (**b**) amplitude and phase images in lock-in thermography [157]; (**c**) defective and non-defective reference regions [155].

PCBs serve as crucial structures for achieving electrical and pneumatic interconnection, signal transmission, mechanical linkage, and support for electronic systems. They also represent the primary failure-prone areas for components, especially in high-frequency and high-voltage circuits. Hence, the detection and maintenance of faults in PCBs are critical due to their complex multi-layered structures, leading to various defects such as layer separation, delamination, breakdown damage, and micro-holes during processing and usage. Conventional defect detection techniques for PCBs encompass visual inspection by human operators and automated optical inspection, X-ray, CT imaging, ultrasound, laser ultrasonics, and terahertz imaging. While manual visual inspection and automated optical inspection are the most common methods, they are limited to detecting visible surface-level defects and cannot guarantee the absence of internal flaws. IRT inspection, as a non-contact measurement method, has gradually found application in the field of PCB fault detection. PCB fault detection methods based on IRT mainly involve three steps: thermal source identification, feature extraction, and thermal pattern recognition [154]. Figure 14a shows 2D and 3D views of the PCBs transient amplitude images. Wang et al. [155] employed laser-induced lock-in thermography to detect various real defects in rigid or flexible PCBs. Phase characteristic images enabled effective detection of delamination defects with a depth of 1.2 mm and micro-hole defects with a depth of 400 µm. The reference regions for both defective and non-defective areas are illustrated in Figure 14c. Experimental results demonstrated that laser-induced thermography is suitable for detecting multiple types of PCB defects. Avdelidis et al. [156] utilized two different integrated pulse thermography systems: thermoscope and echotherm. In both cases, mid-wave infrared cameras were used; a merlin 3–5 µm thermoscope system and a phoenix 3–5 µm echotherm system. Both systems were state-of-the-art portable non-destructive testing and electronic inspection systems with integrated flash heating capability. The results showed that pulse thermography can be used for defect detection in circuit boards (i.e., delamination and/or soldering defects). Cong et al. [157] proposed and utilized optical/thermal fusion imaging technology to inspect PCBs. A semiconductor laser diode with a wavelength of 808 nm was employed as the radiation source. Sample data and images were acquired using a mid-infrared camera. Phase-locked thermal imaging was employed for the study of layered defects in PCBs, as illustrated in Figure 14b. Six different fusion algorithms were applied in the experimental study of image fusion, and four metrics were introduced to evaluate the fusion performance. The experimental results indicate that this fusion technology maintains a high level of accuracy and precision under diverse imaging conditions.

### 4.3. Weld

**Figure 15:**
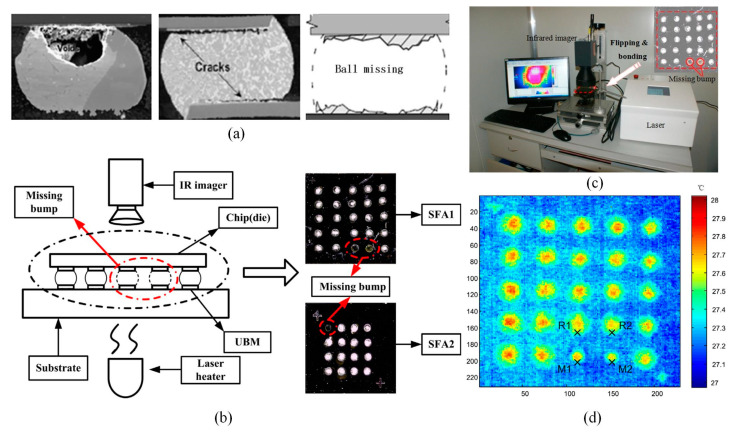
Schematic diagram of welding defect detection: types of weld defects (**a**) [159]; (**b**) schematic of experimental setup and distribution of welds in test samples [162]; (**c**) experimental setup and distribution diagram [161]; (**d**) infrared thermal images of weld defects [163].

Solder joints constitute crucial components on PCBs. Apart from serving as electrical conduits, they also provide mechanical connections between electronic components and the substrate. Solder joints are more susceptible to defects such as cracks, voids, and missing balls, as depicted in Figure 15a [159]. These flaws can adversely affect the performance and lifespan of flip-chip packages, leading to erratic circuit behavior and intermittent instability. This poses significant risks for debugging, operation, and maintenance of circuits. Therefore, the assessment of solder joint integrity holds paramount importance. Presently, conventional non-destructive testing methods such as X-ray, optical inspection, and flying probe testing struggle to effectively detect such welding defects. In contrast, infrared non-destructive testing offers a wide applicability, non-contact measurement, rapid detection, high precision, ease of qualitative and quantitative analysis, as well as convenient observability, presenting a comprehensive set.

Chai et al. [160] proposed an active transient thermography technique for detecting inverted solder balls. When a solder ball is defective, its resistance is significantly higher than that of a normal solder ball, leading to an abnormal temperature. Hence, using thermal image contrast from an infrared sensor, this method detects the presence and location of defective solder balls, primarily void defects and localized cracks. Lu et al. [161] investigated a pulse-phase thermography-based method for identifying solder joint defects. In this approach, the test chip is stimulated with a thermal pulse, and the subsequent transient response is captured using a commercial thermal imaging camera. Thethermal imager was employed to measure the transient response of the test chip under infrared photothermal excitation. The thermal imager, equipped with a micro-lens with a pixel resolution of 25 μm, enhances spatial resolution. The temperature resolution of the thermal imager, utilizing a microbolometer detector, is superior to 80 mK, with a spectral response range of 7.5 to 14 μm, and a frame size of 640 × 480 pixels. Wei et al. [162] developed an intelligent system for detecting solder joint defects using active thermography. Figure 15b illustrates the experimental setup, employing a fiber-coupled semiconductor laser with a central wavelength of 808 nm as the heat source, monitored by the thermal imager. Statistical features were extracted and classified using the M-SVM algorithm. All missing protrusions were identified, achieving the highest recognition accuracy. The results demonstrate that the combination of active thermography and M-SVM is an effective method for intelligent diagnosis of microelectronic packaging solder material defects. He et al. [163] utilized a pulsed laser with a central wavelength of 808 nm to heat the substrate of the test sample SFA1. The sample consisted of 25 solder balls arranged in a 5 × 5 pattern, with protrusion diameters and pitch distances of 500 μm and 1000 μm, respectively. Thermal images of the SFA1 package were acquired using the VH680 infrared imager. The experimental setup is depicted in Figure 15c, while Figure 15d shows the thermal image of the experimental sample SFA1. The matrix was used as the desired output vector, and a transformation function was applied to convert the desired output vector from an index to a vector. A PNN was then established with input vectors, output vectors, and propagation speed as parameters. The results indicate that the infrared detection system based on PNN is effective for defect detection in high-density packaging.

### 4.4. Others

Glass fibers are extensively utilized as reinforcement materials, with glass-fiber-reinforced polymers (GFRP) commonly found in electrical and electronic devices, as well as in numerous components used in our daily lives [12]. Glass fibers present a competitive edge due to their lightweight nature and lower cost compared to other reinforcement materials like carbon fibers [164], showcasing superior properties within composite materials [165]. However, the manufacturing process may incur defects, especially the formation of voids. Fuel cells are essential components in emission-free energy conversion, directly converting chemical energy into electricity. The critical aspect of fuel cell functionality lies in the necessity for all distinct sealing layers to be both electrically insulating and hermetic. The material connecting the two steel interconnect sections of the cells is the glass solder layer, which incorporates artificially induced defects in the form of missing solder of varying diameters.

**Figure 16:**
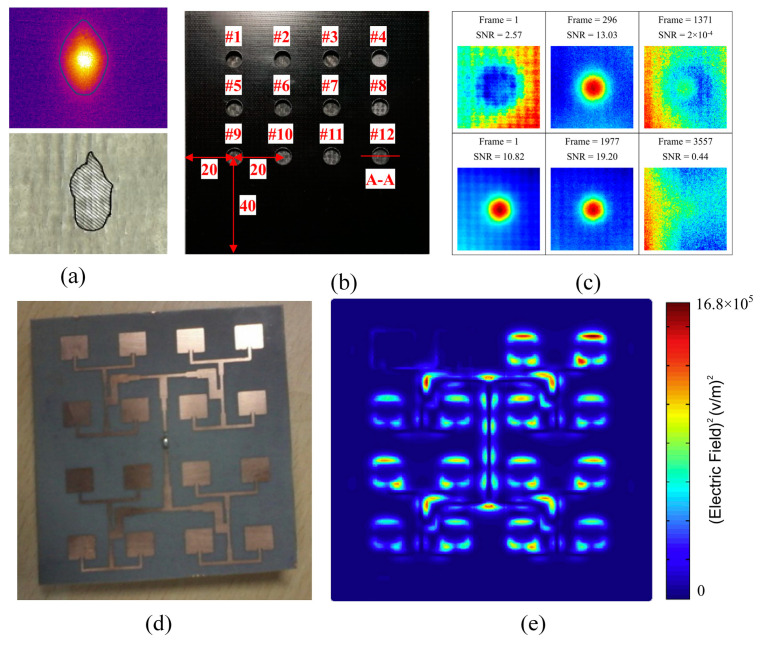
Other applications of IRT: (**a**) thermal and visible images of the sample [166]; (**b**) C/GFRP specimens with artificial flat-bottom holes [171]; (**c**) patch antenna array em-ployed in the experiment [171]; (**d**) phase images of defect #2 in S2 and amplitude images of defect #2 in S2 [168]; (**e**) thermal imaging of defects [168].

Meola et al. [166] conducted an assessment of GFRP under low-energy, low-velocity impact using IRT. They employed a equipped with a quantum well infrared photodetector (QWIP) operating in the 8–9 μm range, with a spatial resolution of 640 × 512 pixels at full frame. For the purpose of comparison, thermal imaging and visible light images of the same sample are presented in Figure 16a. The results demonstrated that non-destructive testing utilizing lock-in thermography could detect manufacturing defects such as uneven resin distribution, porosity, fiber misalignment, and impact damage. Dua et al. [167] introduced a high-depth resolution frequency-modulated thermal wave imaging technique for infrared characterization of GFRP laminates. Each GFRP sample comprised five patches, with a thickness of 2 mm. The selected samples were subjected to experiments using two 1 kW halogen lamps. Thermal distributions of the samples were recorded by an infrared camera at a frame rate of 25 Hz. The results indicated that the layer-wise detection capability of time-correlated coefficient images significantly outperformed the widely used phase-based post-processing methods. Muzaffar et al. [168] proposed a rapid and straightforward method for detecting faults in antenna arrays using infrared thermal imaging. The thermal imager employed was a 14-bit, 320 × 240 resolution mid-wave infrared (MWIR) camera from FLIR. The study demonstrated that IRT could be applied for detecting faulty elements in antenna arrays, with the variation of temperature rise on the absorptive screen being crucial for identifying the faulty components. Figure 16d,e respectively present the sample image and the corresponding thermal imaging of defects. Wei et al. [169] advocated the application of artificial intelligence techniques for automatic processing of infrared images to detect defects within the glass seal layer of solid oxide fuel cells. Three methods were investigated: (1) support vector machine, (2) adaptive enhancement, and (3) U-Net. The results indicated that features extracted from individual thermal profiles might be insufficient for defect identification, while U-Net displayed significant potential in thermal image segmentation. Wang et al. [170] conducted experimental studies on the detection of impact damage in GFRP using pulse radar thermal wave imaging technology. They utilized a high-performance, cooled focal plane infrared imager with a response wavelength of 3.6–5.2 μm and pixel dimensions of 640 × 512. An 808 nm semiconductor laser was used, and various time/frequency domain analysis algorithms were applied to extract features from the thermal image sequences. The thermal image sequence was acquired using an IRT camera, The results showed that the dual-channel orthogonal demodulation algorithm exhibited excellent recognition capabilities for delamination defects in GFRP. Within the specified defect diameter and depth range, it could identify delamination defects with a depth ≥1.70 mm and a diameter-depth ratio (D/H) ≥2.35. By analyzing the signal-to-noise ratio (SNR) of feature images, gong et al. [171] quantitatively evaluated the detection ability of laser bidirectional thermal wave radar imaging (BTWRI) to detect defects of carbon/glass fiber reinforced polymer (C/GFRP). Figure 16b is the sample used in the experiment. By comparing the signal-to-noise ratio of feature images on a frame-by-frame basis, the optimal ACC detection image was obtained. Figure 16c shows the defect phase diagram and amplitude diagram of the sample.

### 4.5. Summary

The advancement of technology has led to increasingly stringent requirements for the quality of electronic components [11]. This chapter provides an overview of the application of IRT in electronic component defect detection from four aspects. Firstly, it introduces the application of IRT in semiconductor chip defect detection. Laser is commonly used as the excitation source, but not all thermal imaging techniques are suitable for detecting defects within semiconductor chip encapsulation. To address this, phase-locked thermography has been developed, which can overcome two limitations of IRT: the inability to differentiate surface and sub-surface features, and the lack of sensitivity. Next, it discusses the application of IRT in PCBs. The structure of PCBs and their relative positions on components are generally fixed. Defect detection in PCBs involves feature matching, and the accuracy of results varies with different parameters. Establishing a neural network in infrared non-destructive defect detection during soldering can enhance the feasibility of defect detection in soldering. In summary, IRT technology, by observing thermal distribution, can identify and address potential thermal issues, faults, or deficiencies in electronic components such as PCBs, chips, soldering, and GFRP. Table 8 provides a summary of the applications of IRT excitation sources in electronic component defect detection and diagnosis.

## 5. Discussion

### 5.1. Algorithmic Detection of PV Panels

**Figure 17:**
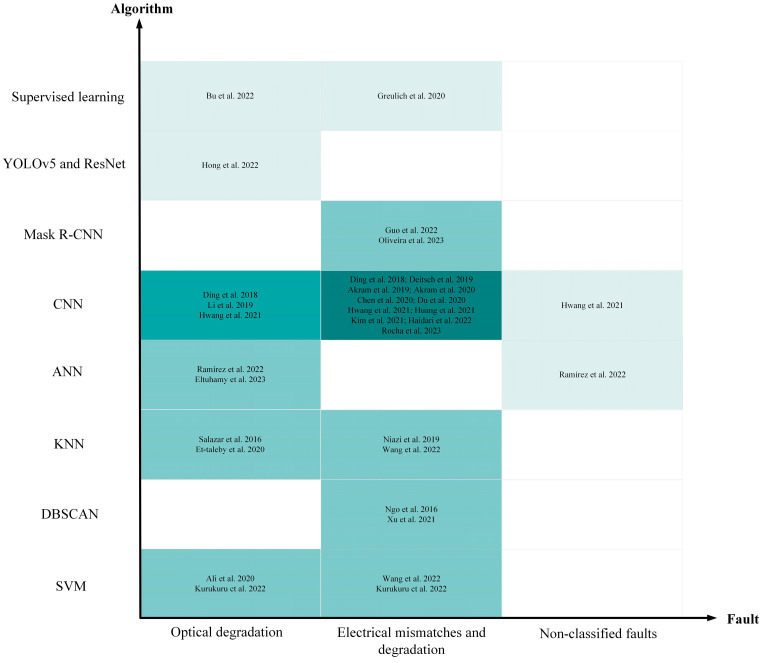
A summary of fault detection in PV panels based on various algorithms and techniques (K-Nearest Neighbours (KNN), You Only Look Once (YOLOv5), Deep Residual Network (ResNet), Adaptive neuro-fuzzy inference system (ANFIS), Naive Bayes (NB), Density-Based Spatial Clustering of Applications with Noise (DBSCAN), Support vector machine (SVM), Artificial neural network (ANN) [83,99,128,134,135,136,138,139,176,177,178,179,180,181,182,183,184,185,186,187,188,189,190,191,192,193,194].

The integration of IRT and deep learning techniques significantly enhances the precision of detecting and diagnosing defects in PV panels [29,172]. This approach typically demonstrates a high level of accuracy in its detection performance, with specific metrics depending on the chosen deep learning model and the quality and scale of the dataset used [173]. The Figure 17 provides a comprehensive overview of the various research studies related to defect detection in PV panels. An analysis of the data highlights several key trends and significant findings in this area. First and foremost, it is evident that research efforts employing CNN as the primary algorithm for PV panel defect detection have been the most prolific. This dominance underscores the efficiency and high defect recognition rates achieved through CNN-based approaches. These neural networks have demonstrated remarkable capabilities in pattern recognition and have significantly advanced the field of PV panel inspection. Furthermore, the integration of cutting-edge deep learning techniques with unmanned aerial vehicles (UAVs) has resulted in a substantial boost in the efficiency of IRT for PV panel inspection [132,174,175].

Additionally, it is essential to acknowledge that earlier studies in this area have been relatively scarce, which emphasizes the rapid advancement and evolving landscape of deep learning’s role in photovoltaic research. In summary, the research landscape in PV panel defect detection is marked by a strong reliance on CNN algorithms for their efficiency and high recognition rates. Additionally, the synergistic use of deep learning and UAVs with IRT has greatly enhanced the speed and effectiveness of PV panel inspection, promising a brighter future for this field.

### 5.2. Excitation Sources of Electronic Industry

In the IRT inspection of PV panels, it is common practice to utilize external natural light sources or indoor lighting, such as sunlight or thermal radiation from the PV cells, as the thermal excitation source [195]. These light sources illuminate the surface of the PV panel, resulting in the absorption of energy by the panel and subsequent temperature elevation. Subsequently, an infrared thermal imaging camera captures the thermal radiation emitted by the PV panel, generating thermal images for the purpose of further analyzing and detecting anomalies or defects in the surface temperature distribution [196]. It is noteworthy that this approach typically obviates the need for additional artificial excitation sources, relying instead on naturally occurring or ambient light sources for the thermal imaging inspection. This inherent advantage enhances the convenience of the detection process and renders it suitable for the monitoring and maintenance of practical PV panels.

In contrast to infrared thermal imaging detection in PV panels, the detection of electronic components differs due to their complex and intricate structures. Often, external excitation is required to induce heating for these electronic components. This allows the thermal radiation of the object under inspection to be captured by the infrared camera, generating a thermal image. Subsequently, the obtained thermal image is subjected to further analysis to diagnose any defects in the specimen. In the infrared thermal imaging detection of electronic components, lasers are commonly used as the excitation source. This preference arises from the fact that lasers do not induce stress concentration or subsequent damage on the surface of brittle materials. The prevalent laser wavelength used for this purpose is 808 nm. Various factors influence the interaction between the laser and the sample surface during laser stimulation. The primary influencing factors encompass laser power, sampling frequency, convective heat transfer, laser beam diameter, spatial resolution, and thermal camera noise. Table 9 summarizes the characteristics of the excitation source for the detection object.

**Figure 18:**
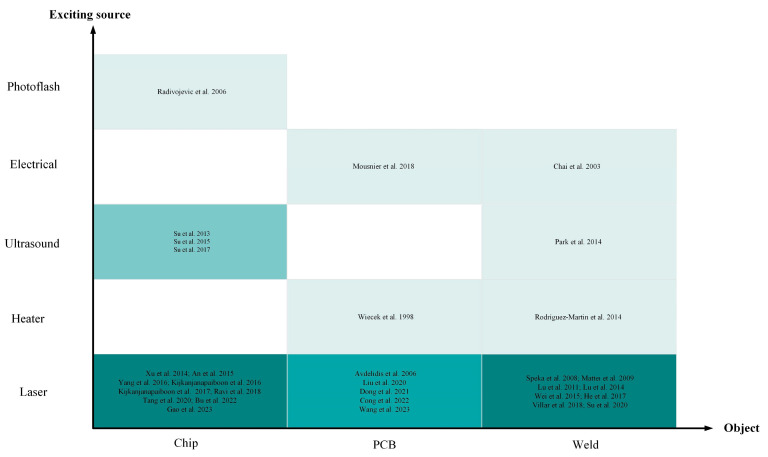
Type of excitation source commonly used in the electronics industry [25,26,145,150,151,152,153,155,156,157,158,159,160,161,162,163,197,198,199,200,201,202,203,204,205,206,207,208,209,210,211,212].

In the last few years, the field of non-destructive testing in the electronics industry has made remarkable progress. Figure 18 shows the types of excitation sources commonly used in the electronics industry in recent years, especially in the field of chips, PCBS, and welding. These excitation sources include lasers, heaters, ultrasound, electricity, and flashlights. As can be clearly seen from the figure, a wide variety of laser sources have been used in past research, and these laser sources show different advantages in different application scenarios. However, recent studies have shown that laser is increasingly used as an excitation source in non-destructive testing in the electronics industry. There are several reasons behind this trend. First, the relatively low cost of the laser makes it the preferred incentive source for many researchers and engineers. Second, the laser is able to cover a large, heated area, which is important when dealing with complex electronic components. Compared with other excitation sources, the laser has a wider heating range and can detect the properties of the target material more comprehensively. In addition, in different practical applications, the researchers found that the wavelength of 808 nm laser is the most commonly chosen laser in the electronics industry in non-destructive testing performance. In general, past and present studies have shown that lasers, as the main excitation source in non-destructive testing in the electronics industry, have the advantages of lower cost, wide heating range, and wavelength. This trend not only reflects the importance of laser technology in the electronics industry, but also provides useful enlightenment for future research and application.

### 5.3. Wavelengths

The application of IRT technology in electronic components not only enables effective detection of defects at the micron level but also facilitates real-time monitoring during the manufacturing process of electronic components such as semiconductors. For instance, in the study conducted by Yang et al. [152], which encompasses data acquisition and processing, the total inspection time for each semiconductor chip is less than 1 s, successfully detecting cracks within a 20 μm range. In summary, IRT technology provides an efficient, non-destructive, and highly accurate method for defect detection in electronic components, enhancing detectability while also serving as a reference for non-destructive testing of similar materials.

**Figure 19:**
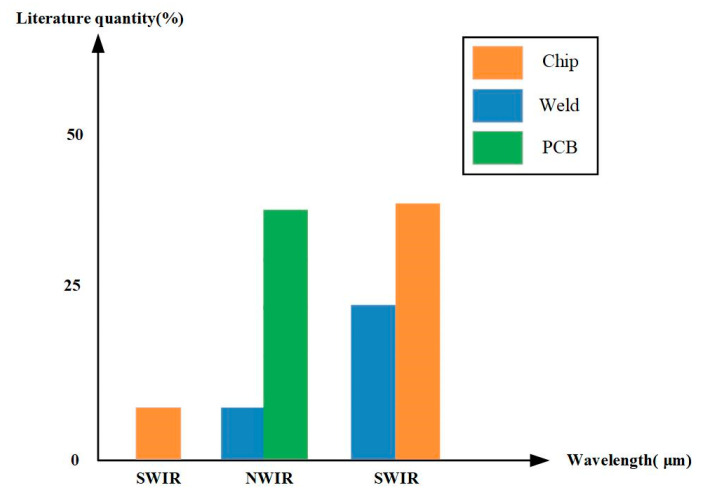
The proportion of the number of references at different wavelengths in the electronics industry.

Figure 19 shows the proportion of different bands in our selected references. It can be seen from the figure that the utilization rate of long-wave infrared and medium-wave infrared in the electronic industry is relatively high. In the non-destructive testing of PCB, only a single mid-infrared wave is used, and its wavelength range is 3 to 5 μm. In the non-destructive testing of chips, long infrared waves with a wavelength range from 7.5 to 14 μm are commonly used, and in the non-destructive testing of welding, both mid-infrared waves and long infrared waves appear. The absorption of infrared radiation by different materials is different, and it can be seen that the choice of wavelength is related to the size, material, structure, and other aspects of the product to be tested.

## 6. Outlooks

The future of active infrared imaging for defect detection in the renewable and electronic industries will be characterized by advancements in excitation sources, improvements in PV panels, widespread adoption in electronics manufacturing, and seamless integration with AI, leading to more efficient, accurate, and cost-effective defect detection solutions. The outlook is given in the following areas:

(1) The future of active infrared imaging for defect detection holds promising developments in excitation sources. Research is expected to focus on more efficient, compact, and versatile excitation methods. Emerging technologies such as advanced lasers and LED arrays may provide more controlled and tailored excitation, enhancing defect visibility [213]. Future research may also delve into multi-modal excitation sources that combine various energy types, such as ultrasound and electromagnetic radiation, with infrared illumination. This fusion of excitation modalities could unlock new possibilities in defect detection by exploiting complementary interactions between materials and different energy sources.
(2) Future research endeavors should prioritize the development and refinement of an expanded array of algorithms tailored for the detection of PV panels irregularities and defects. This emphasis on algorithmic innovation is essential to further enhance the precision and efficiency of PV panel inspection, making it an exciting and crucial avenue for future research. These advanced algorithms should encompass a wide range of imaging techniques and modalities, including IRT, EL, and optical imaging, among others. By diversifying the algorithmic approaches, researchers can effectively address the multifaceted challenges associated with PV panel inspection.
(3) The electronics industry will increasingly adopt active infrared imaging for quality control and defect detection during manufacturing. Active infrared imaging will provide real-time quality control during manufacturing processes. As electronic components are assembled, the integrated infrared sensors will continuously monitor for defects, irregularities, and variations in temperature or electrical performance. This real-time feedback loop allows for immediate adjustments and corrections, reducing the likelihood of defects propagating downstream [214]. Compact and cost-effective infrared imaging equipment will be incorporated into manufacturing lines, facilitating swift and accurate examination of electronic components. This integration will contribute to the reduction of defects, improvement of product dependability, and reduction of production expenditures.
(4) The integration of active infrared imaging with AI will revolutionize defect detection. Machine learning algorithms, particularly deep learning techniques like CNN, will become more adept at recognizing complex defect patterns and distinguishing anomalies from normal operation. AI-driven defect detection systems will be capable of real-time analysis, reducing false positives and improving overall accuracy. Beyond detecting defects, AI can predict when components or systems are likely to fail based on their thermal behavior captured through infrared imaging. This enables predictive maintenance, where machinery and equipment are serviced or replaced before they break down, reducing downtime and costly repairs.

## Figures and Tables

**Table 2:** The most cited review on the application of IRT in PV.

Authors	Year	Citations	Title
Tsanakas et al. [6]	2016	200	Faults and infrared thermographic diagnosis in operating c-Si photovoltaic modules: A review of research and future challenges.
Aghaei et al. [81]	2015	102	Innovative Automated Control System for PV Fields Inspection and Remote Control.
Herraiz et al. [22]	2020	75	Photovoltaic plant condition monitoring using thermal images analysis by convolutional neural network-based structure.
Gallardo-Saavedra et al. [82]	2018	71	Technological review of the instrumentation used in aerial thermographic inspection of photovoltaic plants.
Niazi et al. [83]	2019	68	Hotspot diagnosis for solar photovoltaic modules using a Naive Bayes classifier.

**Table 3:** Annual performance metrics of renewable industry.

Year	Documents	Citations	Average Citations per Document	H-Index
2013	65	1954	30.06	25
2014	96	1782	18.56	23
2015	81	1547	19.1	25
2016	99	1485	15	21
2017	140	3820	27.16	29
2018	99	1482	14.97	22
2019	136	1890	13.9	24
2020	136	2436	17.15	26
2021	124	1400	11.29	19
2022	150	641	4.27	11

**Table 4:** Summary of the combination of IRT and deep learning techniques for defect detection and diagnosis of PV panels.

Algorithms	Authors, Year	Purpose	Findings	Remarks
CNN	Du et al. [99] 2020	To enhance the detection efficiency of Si-PV cells and achieved extensive defect detection and classification of Si-PV cells.	The classification results of traditional classification methods were significantly lower than those of CNN models.	IRT and CNN have significant potential for applications in defect detection and automatic recognition in Si-PV cells.
TPC algorithm	Balasubramani et al. [100] 2020	The TPC algorithm detects discoloration and layering defects on PV panels.	The CF’s fuzzy classifier exhibited superior classification accuracy, resulting in an average classification accuracy improvement of 10%.	The TPC algorithm demonstrates a high level of effectiveness in detecting EVA discoloration and layering defects.
Canny edge detection	Tsanakas et al. [106] 2013	Rapid detection and diagnosis of hotspots in PV modules.	The diagnosed hotspots have been validated against the standard electrical tests for each module, indicating a performance decrease of 9.5% for PV-1 and 9.7% for PV-2, respectively.	This method utilizes qualitative and quantitative data from processed thermal images of two PV arrays, providing easily interpretable results.
CSDM and CNN	Lu et al. [16] 2021	A hybrid algorithm combining CSDM and CNN is employed to study fault detection in PV modules.	The proposed method achieved an impressive recognition accuracy of 99.5%.	The algorithm simplifies a substantial amount of raw measurement data through CSDM and subsequently employs CNN to accurately identify the fault states of PV modules.
GA-DBN	Tao et al. [17] 2020	GA are utilized for diagnosing faults in PV arrays to optimize the DBN.	The GA-DBN method effectively enables diagnostic detection of five operational states in PV arrays, achieving an overall diagnostic accuracy of 95.73%.	Compared to the DBN, SVM, and GA-BP models, this model exhibits higher accuracy in both overall diagnosis and individual fault type diagnosis.
CNN	Manno et al. [18] 2021	Utilizing CNN for the automatic classification of thermal images to identify faults in PV panels.	A dataset consisting of 200 segmented images achieved a 100% accuracy rate used CNN.	The CNN method proves to be an effective tool, enhancing the image classification resolution for remote fault detection issues.
DCNNs	Mellit [111] 2022	Embedded PV module fault detection and diagnosis using IRT and DCNNs.	Two DCNN-based models, namely the fault detection and diagnosis models, achieved an accuracy rate of 99% for fault detection and an accuracy rate of 95.55% for fault diagnosis.	Embedded solutions can detect and diagnose faulty PV modules with acceptable accuracy.

**Table 5:** Early and current authors are conducting research on the infrared detection of PV panels.

Authors	Year	Citations	Title	Remarks
Dincer et al. [121]	2014	29	Polarization Angle Independent Perfect Metamaterial Absorbers for Solar Cell Applications in the Microwave, Infrared, and Visible Regime.	The proposed metamaterial-based solar cell demonstrates high absorption in both the infrared and visible spectra, enhancing the potential for more efficient next-gen solar cells.
Chandel et al. [122]	2015	33	Degradation analysis of 28 year field exposed mono-c-Si photovoltaic modules of a direct coupled solar water pumping system in western Himalayan region of India.	Utilizing thermal imaging technology to identify hotspots and quantifying degradation by measuring PV parameters under indoor and outdoor conditions.
Adams et al. [123]	2015	42	Water Ingress in Encapsulated Inverted Organic Solar Cells: Correlating Infrared Imaging and Photovoltaic Performance.	Utilizing infrared imaging for local, in-situ tracking of humidity-induced performance degradation to predict the lifespan of organic solar cells and modules.
Du et al. [124]	2017	38	Nondestructive inspection, testing and evaluation for Si-based, thin film and multi junction solar cells: An overview.	Non-destructive inspection, testing, and assessment of solar cells and modules.
Addabbo et al. [125]	2017	55	A UAV Infrared Measurement Approach for Defect Detection in Photovoltaic Plants.	Drones can swiftly inspect solar farms, employing this positioning technology for detecting, labeling anomalies, and identifying faulty panels.
He et al. [126]	2018	36	Noncontact Electromagnetic Induction Excited Infrared Thermography for Photovoltaic Cells and Modules Inspection.	The active electromagnetic induction infrared thermal imaging defect detection method has enabled the visual detection of defects in PV cells and modules.
Zefri et al. [127]	2018	48	Thermal Infrared and Visual Inspection of Photovoltaic Installations by UAV Photogrammetry-Application Case: Morocco.	Visual defects, such as cracks, contamination, and hotspots, have been identified in both visual RGB and thermographic inspections.
Akram et al. [128]	2020	80	Automatic detection of photovoltaic module defects in infrared images with isolated and develop-model transfer deep learning.	CNN are used to train an isolation learning model, achieving an average accuracy of 98.67%. Fine-tuning the pre-trained base model through transfer learning on an infrared image dataset increased accuracy to 99.23%.
Du et al. [99]	2020	43	Intelligent Classification of Silicon Photovoltaic Cell Defects Based on Eddy Current Thermography and Convolution Neural Network.	IRT and CNN demonstrate significant potential for defect detection and automatic recognition in Si-PV cells, providing a reliable approach for the research, testing, manufacturing, servicing, and maintenance of Si-PV cells.
Alves et al. [129]	2021	40	Automatic fault classification in photovoltaic modules using Convolutional Neural Networks.	Using cross-validation methods, CNN achieve an estimated accuracy of 92.5% in detecting anomalies in PV modules.

**Table 6:** Annual performance metrics of electronic industry.

Year	Documents	Citations	Average Citations per Document	H-Index
2013	45	680	15.11	13
2014	52	483	9.29	10
2015	89	856	9.62	17
2016	47	493	10.49	11
2017	29	483	16.66	11
2018	58	650	11.21	13
2019	49	514	10.49	12
2020	53	531	10.02	14
2021	58	587	10.12	10
2022	55	287	5.22	9

**Table 7:** Early and current authors are conducting research on the infrared detection of electronics industry.

Authors	Year	Citations	Title	Remarks
Jadin et al. [143]	2012	19	Infrared Image Enhancement and Segmentation for Extracting the Thermal Anomalies in Electrical Equipment	The segmentation performance of infrared images is improved by image enhancement method which adjusts the image intensity.
Rogalski et al. [144]	2013	28	Semiconductor detectors and focal plane arrays for far-infrared imaging	The progress of far infrared and submillimeter wave semiconductor detector technology in focal plane array in recent 20 years is introduced.
Xu et al. [145]	2014	18	Using active thermography for defects inspection of flip chip	The feasibility of the flip chip defect detection method based on active thermal imaging is proved.
Daimon et al. [146]	2016	98	Thermal imaging of spin Peltier effect	The combination of spin Peltier effect and lock-in thermography technology provides a new direction for spintronics applications.
Christensen et al. [147]	2018	75	The OSIRIS-REx Thermal Emission Spectrometer (OTES) Instrument	It provides precise moving mirror control and infrared sampling at 772 Hz and minimizes surface reflection.
Aragon et al. [148]	2020	36	A Calibration Procedure for Field and UAV-Based Uncooled Thermal Infrared Instruments	A new calibration method of ambient temperature correlation for a variety of uncooled thermal infrared radiometers is proposed, which significantly improves the measurement accuracy.
Yu et al. [149]	2020	29	Low-Cost Microbolometer Type Infrared Detectors	The advantages of pixel size reduction are significant.

**Table 8:** Applications of IRT excitation sources in electronic component defect detection and diagnosis.

Exciting Source	Authors, Year	Purpose	Fundings	Remarks
Linear frequency modulation (LFM) laser	Tang et al. [150] 2020	To perform non-destructive testing on surface/sub-surface damage during the production process of semiconductor silicon wafers.	It could effectively identified microcracks of 10 μm, with a theoretical minimum detectable temperature difference less than 0.401 K.	In theory, microcracks with a width of 10 μm can be detected.
Barker-code laser	Bu et al. [151] 2022	Conducting non-destructive testing on semiconductor silicon wafers.	The BCLIT technology enhanced the signal-to-noise ratio and defect detectability.	Providing theoretical basis and operational reference for BCLIT technology in detecting microcrack defects in semiconductor silicon wafers.
Multi-spot laser	Yang et al. [152] 2016	Real-time inspection during the process of semiconductor chip manufacturing.	Successfully detected cracks within a range of 20 μm.	The MLLT system can be further developed into a standalone system for semiconductor manufacturing facilities.
Line laser	An et al. [26] 2015	To conduct instantaneous detection of surface cracks in semiconductor chips during actual manufacturing.	Successfully conducted visual inspection of cracks in semiconductor chips with widths ranging from 28–54 μm.	Expanding from chip-level to wafer-level for more efficient and faster detection.
Semiconductor laser diode (808 nm)	Wang et al. [155] 2023	To conduct research on the multi-type defect detection of multi-layered complex structured PCBs.	Effective detection of PCBs delamination defected with a depth of 1.2 mm and micro-hole defected with a depth of 400 μm.	Laser-induced lock-in thermography is suitable for detecting defects in the complex, multilayered structure of PCBs.
Laser (808 nm)	Xu et al. [145] 2014	To investigate a thermography-based active method for solder joint inspection.	The detection method based on active thermal imaging was effective for identifying missing protrusions in flip-chip packages.	Further research is needed to differentiate subtle defects in flip-chip packaging.
IR lamp	Lu et al. [161] 2011	To investigate the defect identification method for solder joints based on pulse-phase thermography.	The phase profilometry technique employed phase difference can characterize missing solder bumps defects in high-density packaging.	The detection method based on PPT is effective in identifying missing protrusions in high-density packaging.
Fiber-coupled semiconductor laser	Wei et al. [162] 2015	To develop an intelligent system utilizing active thermal imaging technology for detecting solder joint defects.	Resolved the issue of small sample sizes in solder defect detection, achieving the highest level of identification accuracy.	The combination of active thermography with M-SVM is an effective approach for intelligent diagnosis of solder defects in microelectronic packaging.

**Table 9:** Summary of characteristics of the excitation source for the detection object.

Detection Object	Excitation Source	Purpose	Characteristic	Remarks
PV panels	Thermal radiation [21]	The PV components exhibit abnormal temperature distribution at faulty and damaged areas.	IRT is characterized by its non-destructive testing technology for safety.	The use of machine learning methods based on IRT has been proven to have high accuracy (up to 99%) in PV detection and fault diagnosis.
Chip	Electromagnetic waves [145]	Active thermal imaging for solder joint inspection.	Inverted chips are heated by a non-contact heating source.	The active thermal imaging detection method can effectively identify missing bumps in inverted chips.
Weld	Thermal radiation [197]	Thermal imaging testing is used for the detection of sub-surface cracks in welding.	Thermal data is used to study the cooling trends in both defective and non-defective areas.	After detecting defects, they can be differentiated based on their morphology.
PCBs	Thermal radiation, electromagnetic waves [155]	Laser-induced phase-locked thermography technology is used to detect various defects in PCBs.	It can accurately identify defects with flat-bottom holes at depths of 0.2 mm and 0.6 mm.	Laser-induced lock-in thermography is suitable for detecting various types of defects in multi-layer and complex structured PCBs.
